# Genomic signatures highlight stress-response evasion and translational tuning as key drivers of *Escherichia coli* adaptation to fluorinated tryptophans

**DOI:** 10.1039/d6sc05637g

**Published:** 2026-08-21

**Authors:** Christin Treiber-Kleinke, Jana Madeleine Göller, Justin Liba, Michael Chun-Hin Wong, Silver Anthony Wolf, Allison Ann Berger, Torsten Semmler, Nediljko Budisa, Beate Koksch

**Affiliations:** a Institute of Chemistry and Biochemistry – Organic Chemistry, Freie Universität Berlin Berlin Germany beate.koksch@fu-berlin.de; b Chemical Synthetic Biology Group, Department of Chemistry, University of Manitoba Winnipeg MB R3T 2N2 Canada nediljko.budisa@umanitoba.ca; c Genome Competence Centre (MF1), Robert Koch Institute Berlin Germany; d Coordination Integrated Genomic Surveillance (IGS), Robert Koch Institute Berlin Germany

## Abstract

Fluorinated amino acids profoundly perturb cellular physiology because they enter the proteome while differing from their natural counterparts in subtle but functionally important ways. Here we investigated how *Escherichia coli* adapts to the biosynthesis and proteome-wide incorporation of fluorinated tryptophans derived from 4-, 5-, 6-, and 7-fluoroindoles using adaptive laboratory evolution (ALE). Whole-genome sequencing of independently evolved populations revealed convergent adaptive solutions. All 6- and 7-fluoroindole lineages acquired disruptive mutations in the stringent starvation regulator (*sspA*), effectively attenuating stress signaling and allowing continued expression of housekeeping functions despite proteotoxic pressure. In parallel, recurrent mutations in tryptophanyl-tRNA synthetase (*trpS*), which charges tRNA^Trp^ with tryptophan, pointed to translational tuning consistent with improved handling of fluorinated substrates, with Q27P emerging most prominently. In several 6-fluoroindole populations, additional defects in *mutS*, involved in DNA mismatch repair, allowed replication errors to accumulate, generating transient hypermutator states that accelerated evolutionary exploration but were not required for successful adaptation. Reconstruction experiments confirmed that loss of stringent response control and altered TrpRS function together increased fitness during fluorotryptophan incorporation. Together, these results suggest that adaptation does not appear to primarily rely on establishing a fundamentally new fluorine-based biochemistry, but rather on adjustment of stress-response regulation and translational control. More broadly, this work establishes a general framework for understanding, and ultimately engineering, microbial adaptation to non-natural metabolites through targeted modification of regulatory and translational control nodes rather than metabolic redesign.

## Abstract 2

Genomic analysis of evolved *E. coli* lineages reveals adaptation to fluorinated tryptophans through retuning of cellular control systems.

## Graphical abstract

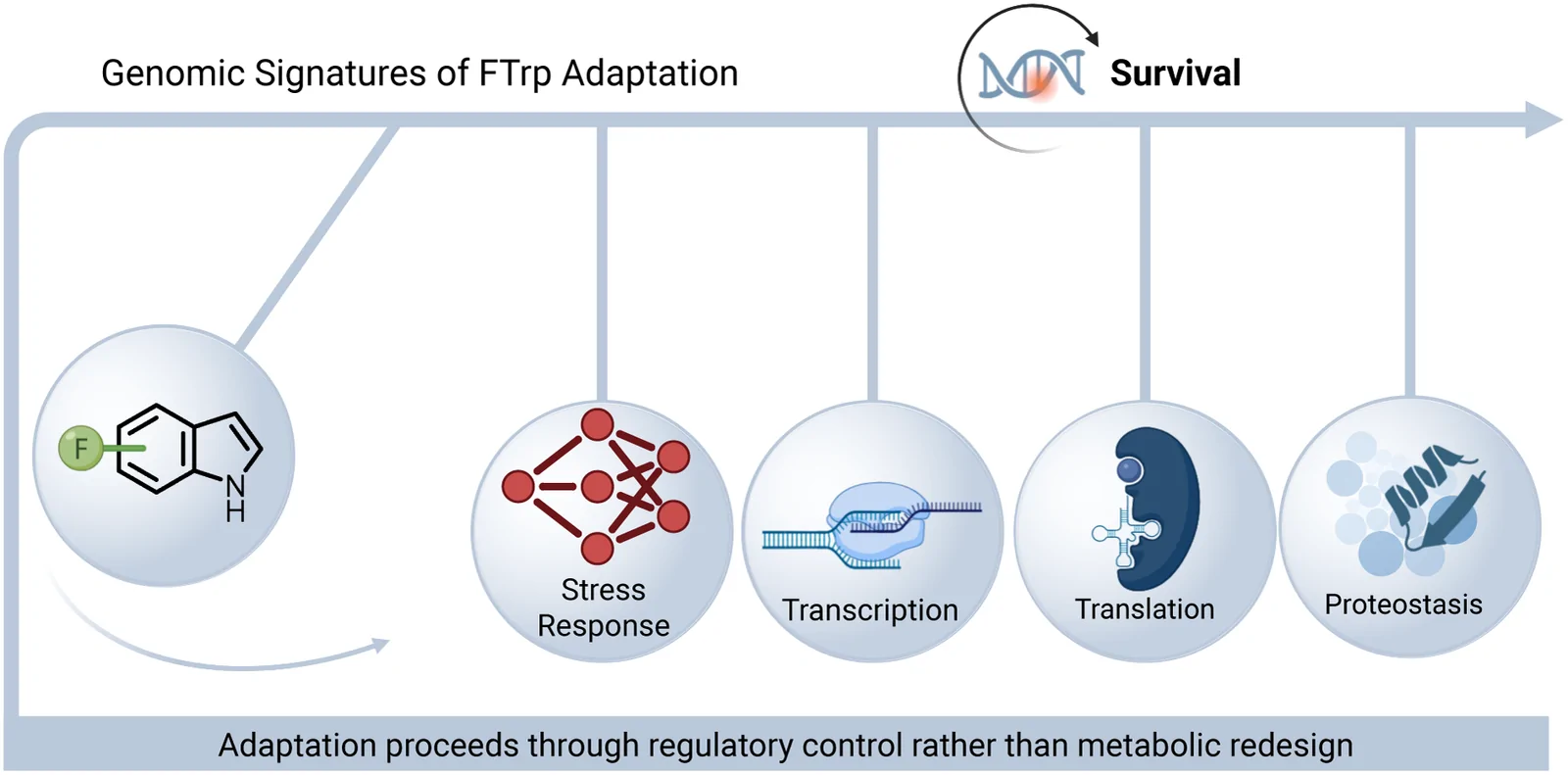

## Introduction

The interaction of microorganisms with organofluorine compounds is of increasing environmental and biotechnological relevance. Fluorinated chemicals, including per- and polyfluoroalkyl substances (PFAS) and fluorinated pharmaceuticals, are chemically persistent and frequently toxic to living systems. Understanding how cells tolerate or respond to such compounds is therefore important both for bioremediation strategies and for engineering microbial production platforms that operate in the presence of fluorinated metabolites.^1–5^

Fluorinated amino acids provide a particularly informative model system because they closely resemble canonical metabolites yet perturb cellular physiology when incorporated into proteins.^6–8^ Substitution of hydrogen by fluorine alters polarity, sterics, and electronic properties, frequently impairing protein folding and function.^9,10^ Consequently, exposure to fluorinated amino acids imposes a combined metabolic and proteotoxic stress on the cell. Studying how microorganisms adapt to this condition allows observation of the physiological mechanisms that maintain proteome functionality under chemical perturbation.

Adaptive laboratory evolution (ALE) is a powerful approach to investigate such processes. By serially propagating microorganisms under defined selective pressure, beneficial mutations accumulate and can subsequently be identified by whole-genome sequencing, linking genotype to fitness. ALE has been widely applied to uncover mechanisms of metabolic adaptation, stress tolerance, and regulatory rewiring.^11–14^

The incorporation of fluorinated amino acids into living systems has been investigated for several decades. Early feeding experiments in the 1960s demonstrated partial incorporation of fluorinated analogs into proteins but also revealed strong toxicity.^15–17^ Later, Wong and coworkers generated a *Bacillus subtilis* strain capable of replacing tryptophan with 4-fluorotryptophan (4FTrp), establishing that extensive substitution is biologically possible.^18^ Subsequent ALE experiments in *E. coli* confirmed high-level exchange of tryptophan by 4FTrp, although residual natural tryptophan remained present.^19^ Later studies extended these observations to additional regioisomers such as 5- and 6-fluorotryptophan (5FTrp, 6FTrp).^20,21^

**Fig. 1:**
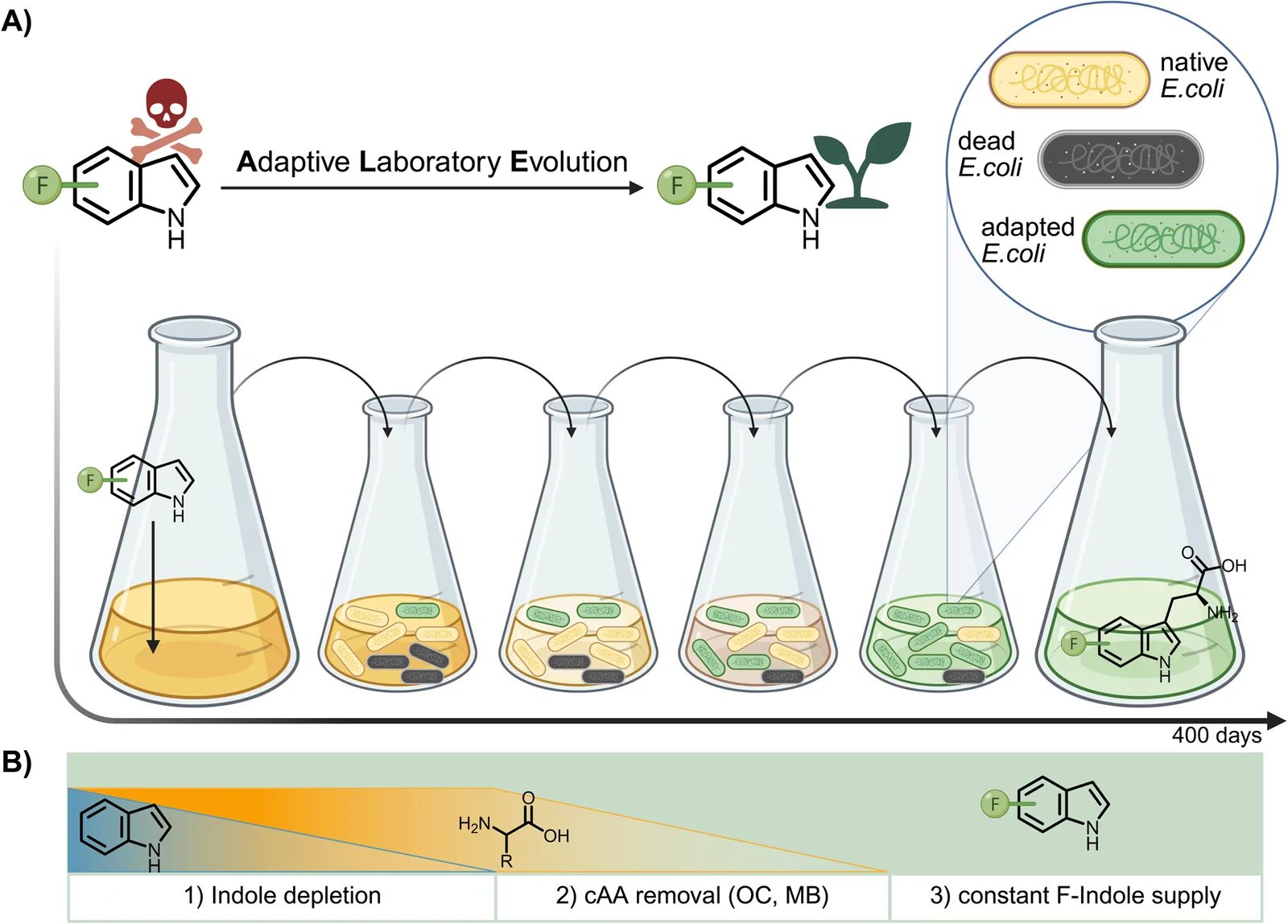
Adaptive laboratory evolution toward growth on fluorinated tryptophan precursors. (A) Serial passaging scheme illustrating progressive population replacement during ALE. Cells exposed to fluoroindole (Fi) initially show growth inhibition, followed by enrichment of adapted subpopulations over successive transfers. (B) Nutrient regime during selection. The medium contained constant Fi while indole and canonical amino acids (except tryptophan) were gradually withdrawn, forcing growth to rely on Fi-derived fluorotryptophan.

Recent work demonstrated that tryptophan-auxotrophic *E. coli* can be evolved to grow on fluoroindole precursors that are converted intracellularly into fluorinated tryptophans by tryptophan synthase (Fig. 1A). These adapted strains achieve global substitution of tryptophan in the proteome.^22,23^ Multi-omics analyses indicated extensive cellular rearrangements, including regulatory changes, membrane adaptations, and enhanced protein quality control, suggesting that adaptation involves system-level physiological responses rather than simple modification of the translation machinery.

However, the genetic mechanisms linking mutation patterns to restored cellular fitness remain incompletely understood, particularly for the 6- and 7-fluorotryptophan isomers. While previous studies established the feasibility of adaptation, they did not resolve which mutations directly enable growth and which represent secondary evolutionary trajectories.

Here we present comparative genomic analyses of multiple independently evolved *E. coli* populations adapted to fluoroindole-derived fluorotryptophans.^22,23^ We identify three recurring adaptive mechanisms: attenuation of stress signaling, tuning of aminoacyl-tRNA charging, and the transient emergence of hypermutator phenotypes. Reconstruction experiments demonstrate that growth restoration results primarily from regulatory bypass and translation retuning rather than from the establishment of a dedicated fluorine-dependent metabolism. These findings define the physiological strategy by which cells preserve proteome functionality during global incorporation of a chemically perturbed amino acid.

## Results

### Overview of the adaptive evolution experiments

To relate genomic variation to fluorotryptophan adaptation, we analyzed whole-genome sequencing data from six ALE experiments performed with mono-fluorinated indoles (4Fi–7Fi) and two non-fluorinated controls (indole and tryptophan). The 4Fi and 5Fi genomic datasets were previously reported by Agostini *et al.* (2021),^22^ whereas the adaptation experiments for 6Fi and 7Fi lineages were reported by Treiber-Kleinke *et al.* (2024)^23^ without accompanying genome-level analysis. Here, we build on these studies by providing the first genomic analysis of the 6Fi/7Fi-adapted populations and by re-analyzing the published 4Fi/5Fi genomes within the same population-genomic framework, including mutation-rate estimates, coding-sequence selection signatures, nucleotide-substitution spectra, and gene-level convergence across all four fluorotryptophan isomers. This comparative design allows us to move beyond lineage-specific descriptions and ask whether adaptation to distinct fluorotryptophan isomers follows shared evolutionary routes. The combined dataset comprises the 4TUBX and 5TUBX trajectories, three independent 6Fi lineages (6TUBX-OC, 6TUBX-MB4, 6TUBX-MB3), two 7Fi lineages (7TUBX-OC, 7TUBX-MB), and two control populations (W-TUBX, Ind-TUBX). Lineage nomenclature follows the convention Z-TUBX-OC/MB, where “Z” denotes the Trp-source (Ind, Trp, 4Fi–7Fi), “X” the passage number, and “OC/MB” the amino-acid removal strategy.

All experiments followed the same three-phase selection logic (Fig. 1B). In phase 1, cultures were gradually weaned off indole, which initially served as growth supplement. Phase 2 consisted of the progressive removal of the 19 canonical amino acids (cAAs) to enforce exclusive reliance on the fluorinated indole analog. Two cAA removal strategies were employed: (1) OC (overall concentration), reducing the total concentration of all cAAs stepwise, and (2) MB (metabolic blocks), removing amino acids according to their metabolic origin.^24^ The 6Fi and 7Fi ALEs therefore bifurcated into OC and MB sublineages, whereas the earlier 4Fi/5Fi experiments used only the MB strategy. Phase 3 marks the stage of exclusive growth on the respective fluorinated indole analog (or on indole/Trp for the positive controls). The resulting lineage relationships are summarized in Fig. S1.

From each lineage, multiple population samples representing key stages of the adaptation trajectory were subjected to whole-genome sequencing (typically 4–8 samples per lineage; Table S1, SI method 1). Because only bulk-population samples were sequenced, the resulting mutation calls reflect allele frequencies within evolving populations rather than single-clone genotypes. Sequencing was performed at high depth (100X median coverage, avg. of 4.8 mio reads per sample), providing reliable detection of fixed and high-frequency variants. Single nucleotide polymorphism (SNP)-calling was used to reconstruct the mutational trajectories and to identify genetic signatures associated with successful adaptation to fluorinated indoles.

### Mutational landscapes and mutation spectra

**Fig. 2:**
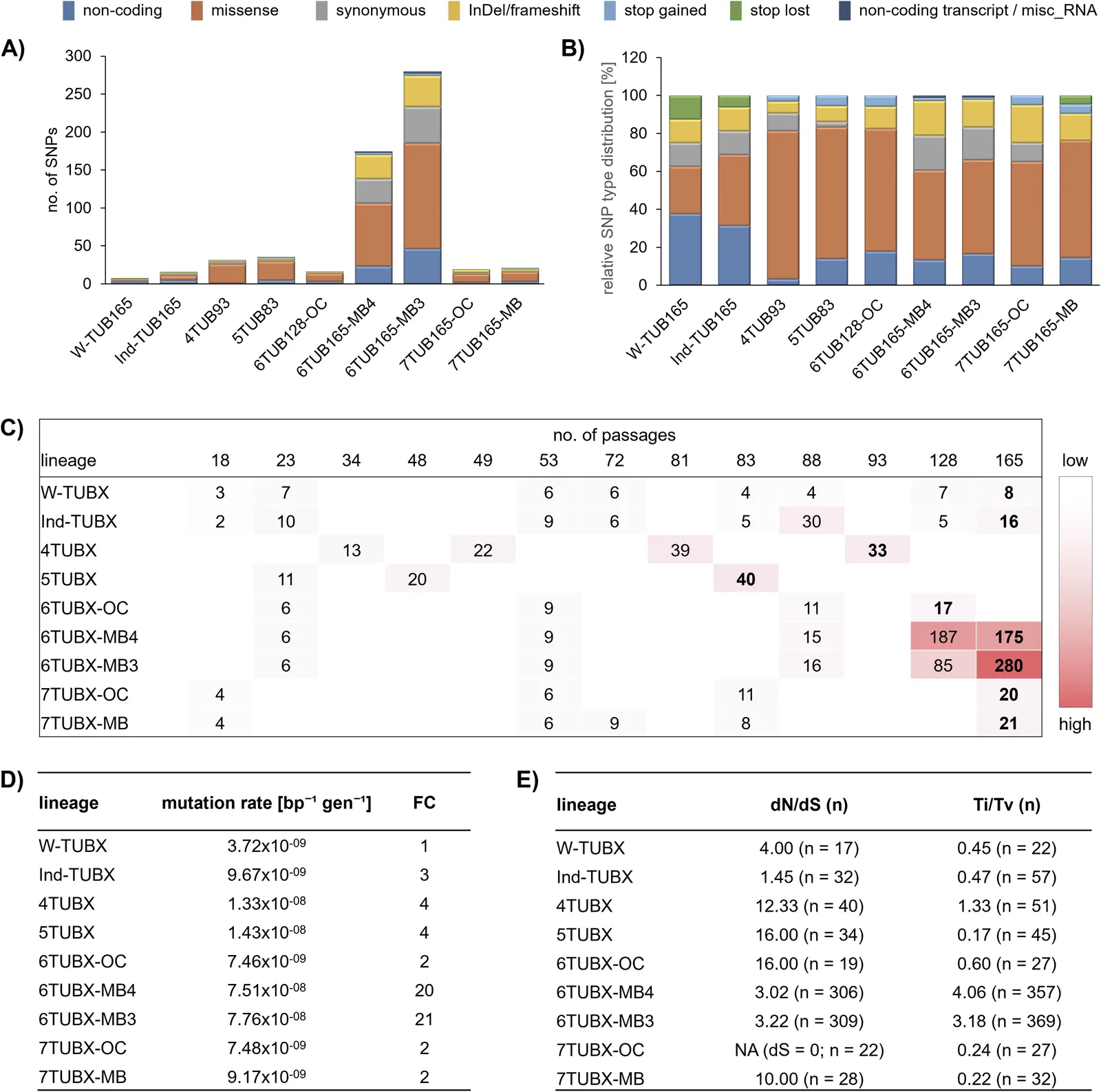
Mutational landscapes, temporal mutation accumulation, mutation rates, and mutational spectra during Fi adaptation. (A) Total number of mutations detected in final populations and (B) relative distribution of mutation classes in final populations; bars are segmented by mutation class (non-coding, missense, synonymous, indel/frameshift, nonsense, non-coding RNA). (C) Heatmap of mutation counts across sequenced passages. Columns indicate passages, rows indicate lineages; color intensity denotes mutation load from white/light pink (low) to red (high). (D) Lineage mutation rates (base^−1^ generation^−1^) and fold change relative to the tryptophan control (W-TUBX), calculated from mutations across all sampled passages. (E) Mutational spectra per lineage shown as dN/dS and transition/transversion (Ti/Tv) ratios derived from lineage mutation sets; *n* denotes coding sequence (CDS) mutations for dN/dS and all SNPs for Ti/Tv.

Across the combined dataset, mutation load differed substantially between lineages (Fig. 2A–C and Table S2). The control populations accumulated 8 (W-TUB165) and 16 (Ind-TUB165) mutations, whereas most fluoroindole-adapted populations contained 17–40 mutations. The low mutation burden in the Trp control is expected given its growth under the most native-like conditions, while the indole control required *de novo* tryptophan biosynthesis. In contrast, the two 6Fi-MB lineages carried markedly elevated mutation counts (175 and 280 mutations), indicative of hypermutator behavior.

Despite large differences in mutation load (Fig. 2A), the relative composition of mutation types was broadly similar across adapted populations (Fig. 2B). Whereas the control populations contained a higher proportion of non-coding mutations and fewer missense substitutions, the latter type dominated in all Fi-adapted strains (50–78% of all mutations), while synonymous substitutions, small indels (insertion-deletion)/frameshifts, stop-gained and stop-lost variants, and mutations in non-coding regions were less frequent. Notably, even the hypermutator 6Fi-MB lineages retained a mutation type composition comparable to non-hypermutator Fi-adapted strains.

In the 4Fi- and 5Fi-adapted populations, most mutated genes were affected only once. The 6Fi- and 7Fi-adapted populations displayed repeated mutations in the same loci across independent lineages, consistent with parallel adaptation. Mutations were distributed across the genome without detectable clustering (Fig. S2).

### Genomic mutation dynamics and evolutionary signatures

Temporal mutation dynamics across the integrated lineage set revealed three distinct evolutionary modes across all lineages (Fig. 2C). Control populations showed limited mutation accumulation, with the W-TUBX control accruing 8 mutations, corresponding to an experimental baseline mutation rate of 3.7 × 10^−9^ per base per generation, whereas the Ind-TUBX control accumulated 16 mutations, likely reflecting the additional metabolic burden of *de novo* tryptophan biosynthesis. Although these estimates exceed reported wild-type *E. coli* mutation rates of approximately 2.2–2.6 × 10^−10^ per base per generation,^25,26^ they provide the experimental baseline for serial passaging under non-selective conditions and indicate that passaging alone did not induce pronounced unspecific genomic drift. Fluoroindole-exposed lineages 4TUBX, 5TUBX, 6TUBX-OC, 7TUBX-OC, and 7TUBX-MB showed moderate mutation accumulation (17–40 SNPs), ranging from control-like levels to modestly elevated mutation loads, consistent with stepwise adaptation in non-hypermutator backgrounds. A markedly different pattern emerged in 6TUBX-MB3 and 6TUBX-MB4, which experienced abrupt mutational bursts between passages 88 and 128, accumulating 175 and 280 mutations, respectively. This marked shift indicates the emergence and rapid population-wide spread of a hypermutator genotype (selective sweep), most plausibly caused by loss-of-function in the mismatch-repair (MMR) gene *mutS*, a known driver of sudden mutation rate elevation in experimental evolution.^27,28^

Lineage-level mutation rates and fold-changes (FC), calculated from mutations aggregated across all sequenced time points, differed markedly between evolutionary regimes (Fig. 2D and Table S3, SI method 2). The tryptophan control W-TUBX showed a low baseline rate (3.72 × 10^−9^ per base per generation), while the indole control exhibited a modest increase (9.67 × 10^−9^; FC = 3), both matching wild-type *E. coli* mutation dynamics.^25^ Fluoroindole-adapted non-hypermutator lineages displayed moderately elevated rates (7.48 × 10^−9^–1.43 × 10^−8^; FC = 2–4), consistent with gradual adaptation without global mutation-rate deregulation. In contrast, the 6TUBX-MB3 and 6TUBX-MB4 lineages showed strongly increased lineage-averaged mutation rates (7.51 and 7.76 × 10^−8^; FC = 20 and 21), consistent with the emergence of MMR-deficient hypermutator genotypes.

To further characterize the underlying mutational processes, we quantified dN/dS ratios (non-synonymous to synonymous substitution rate),^29^ which indicate the balance between protein-altering and silent substitutions, and Ti/Tv spectra (transition/transversion ratios), which reveal biases in the types of nucleotide changes accumulating in each population (Fig. 2E and Table S4, SI method 2). Both controls accumulated relatively few SNPs (*n* = 17 and 32), limiting the statistical power of dN/dS estimates. The W-TUBX control remained close to neutral expectations (dN/dS = 1.45), consistent with limited selective pressure under control conditions, whereas the elevated Ind-TUBX value (dN/dS = 4.00) should be interpreted cautiously given the small number of CDS mutations. Their Ti/Tv values of 0.45 and 0.47 likewise fall within the range expected from stochastic variation when mutation numbers are low. In contrast, the fluoroindole-exposed but non-hypermutator lineages 4TUBX, 5TUBX, 6TUBX-OC and 7TUBX-MB exhibited markedly elevated dN/dS ratios (10–16), indicating a strong excess of non-synonymous substitutions and recurrent protein-level adaptation. These lineages also displayed low Ti/Tv values (<1 for 5TUBX, 6TUBX-OC and 7TUBX-MB, and 1.33 for 4TUBX), signifying transversion-enriched spectra reported under low to mild stress conditions.^30^ Although these signatures suggest adaptive remodeling occurring against a stress-driven mutational background, their reliability remains limited by moderate mutation counts (*n* = 19–51). One lineage (7TUBX-OC) lacked synonymous substitutions (dS = 0; *n* = 22), precluding dN/dS estimation; its low SNP burden and Ti/Tv value (0.24) nevertheless indicate limited adaptive progression and pronounced stress-induced transversion mutagenesis.

A fundamentally different spectrum was observed in the hypermutator populations. 6TUBX-MB3 and 6TUBX-MB4 accumulated by far the largest numbers of mutations (*n* > 300) and showed strongly elevated Ti/Tv ratios (3.18 and 4.06), a transition-enriched signature characteristic of mismatch-repair deficiency, particularly *mutS* or *mutL* loss;^28,31^ and suggestive of stress-induced mutagenesis, potentially involving error-prone DNA polymerases.^32^ Their dN/dS ratios (3.22 and 3.02) are typical of mutator backgrounds, where the rapid accumulation of mutations can overwhelm purifying selection and make genome-wide sequence changes appear more neutral despite ongoing adaptation. These observations strongly support the emergence of MMR-deficient genotypes that increase transition mutagenesis while reducing the visibility of selection on protein-coding changes.

Together, the patterns emerging from mutation accumulation, mutation-rate estimates, and mutational spectra consistently delineate three evolutionary regimes: (1) near-neutral background mutagenesis in the control populations, which remained genomically stable and showed wild-type mutation rates; (2) adaptive, stress-associated mutagenesis in non-hypermutator fluoroindole lineages, characterized by moderate mutation accumulation, elevated mutation rates, transversion-enriched spectra, and high dN/dS ratios indicative of recurrent protein-level adaptation; and (3) mismatch-repair-deficient hypermutation in the 6TUBX-MB lineages, marked by abrupt mutation bursts, >10-fold rate increases, and strongly transition-biased spectra. These regimes illustrate how fluoroindole toxicity can drive both targeted adaptive remodeling and, in some cases, the rise of mutator genotypes that fundamentally reshape the mutational landscape. The reliability of these signatures scales with mutation count, with the hypermutator lineages providing high-confidence estimates, whereas datasets based on few SNPs require more cautious interpretation, in line with the reduced sensitivity and interpretability of dN/dS under shallow divergence and limited substitution counts.^33^

### Genomic variation across ALE lineages

Whole-genome sequencing revealed heterogeneous mutation burdens across the nine ALE trajectories, ranging from few variants in non-hypermutator backgrounds to extensive diversification in hypermutator lineages. Comprehensive variant lists and descriptions, including the Human Genome Variation Society (HGVS) nomenclature used throughout (*e.g.*, “c” for coding DNA changes, “p” for protein-level effects and “>” for substitutions), are provided in the SI (SI extended results, Tables S6–S11). Although lineage-wise profiles differed substantially, several genes and pathways appeared repeatedly mutated, suggesting convergent adaptive solutions to fluoroindole exposure. To move from lineage-specific mutation profiles to system-level insight, we integrated mutations across all lineages using an UpSet intersection analysis (Table S5, SI method 2), which captures higher-order overlaps that cannot be extracted from lineage-specific summaries. This analysis revealed a defined set of recurrently mutated genes constituting the core adaptive response to fluorinated tryptophans.

### Intersecting mutation profiles identify convergent adaptive targets of Fi adaptation

**Fig. 3:**
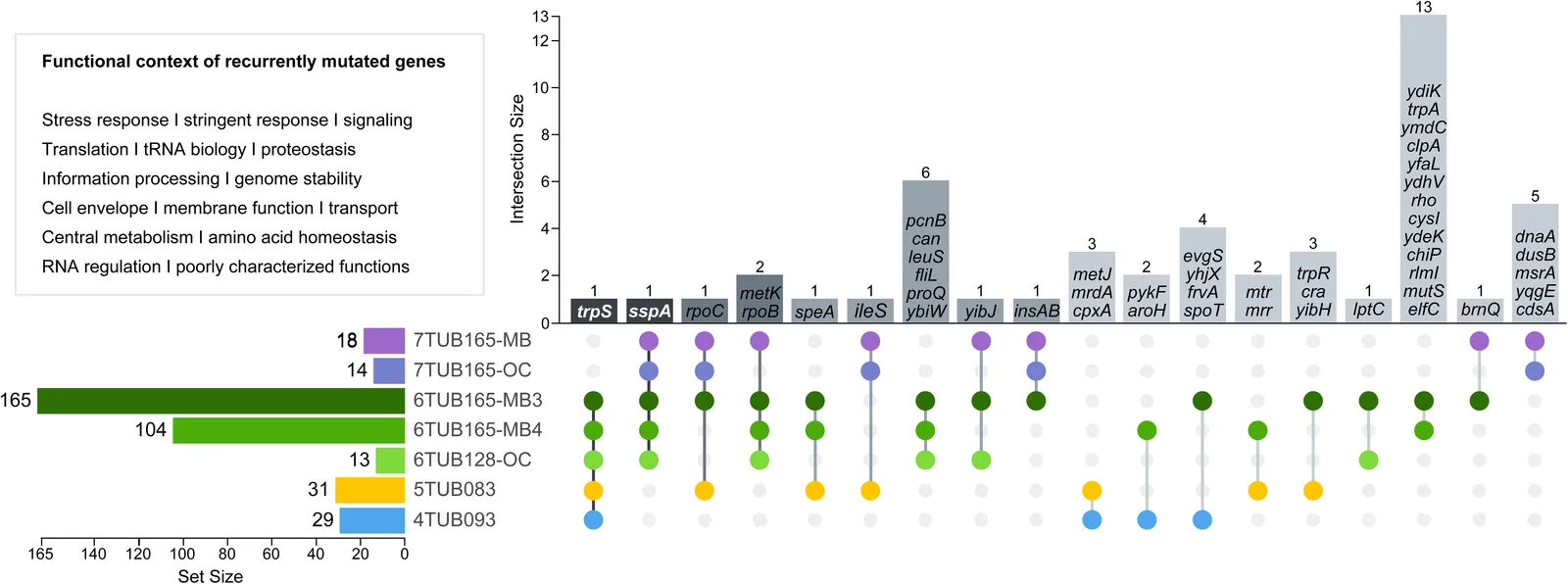
Functional convergence of recurrently mutated genes across independent ALE lineages. UpSet plot showing intersections of genes mutated in multiple evolved lineages. Mutations include endpoint variants and variants classified as fixed due to persistence across sequential passages (Table S5). Bars indicate the number of shared genes; connected dots indicate lineage combinations. Mutated genes were grouped into broad, manually curated functional modules based on Clusters of Orthologous Genes (COG)-like categories and annotated *E. coli* gene functions to aid biological interpretation. Stress response, stringent response and signal transduction: *sspA*, *spoT*, *cpxA*, *evgS*. Translation, tRNA biology and proteostasis: *trpS*, *ileS*, *leuS*, *rlmI*, *dusB*, *clpA*, *msrA*. Genetic information processing and genome stability: *rho*, *rpoB*, *rpoC*, *dnaA*, *mutS*, *mrr*, *insAB-1*. Cell envelope, membrane function and transport: *mrdA*, *lptC*, *chiP*, *mtr*, *brnQ*, *frvA*, *yhjX*, *yfaL*, *ydhV*, *cdsA*, *elfC*, *fliL*. Central metabolism and amino acid homeostasis: *trpA*, *cra*, *trpR*, *metK*, *metJ*, *speA*, *pykF*, *aroH*, *cysI*, *can*. RNA regulation and poorly characterized functions: *pcnB*, *proQ*, *ybiW*, *yibJ*, *yibH*, *ydiK*, *ymdC*, *ydeK*, *yqgE*.

UpSet analysis revealed extensive parallel mutation patterns across independent fluoroindole adaptation experiments (Fig. 3). This convergence occurred despite large differences in mutation load between lineages, including hypermutator strains (6TUB165-MB3, 6TUB165-MB4) and non-hypermutators. Such parallelism is a hallmark of adaptive driver mutations in ALE experiments.^34,35^ The strongest convergent signals occurred in *trpS* (tryptophanyl-tRNA synthetase, TrpRS) and *sspA* (stringent starvation protein A, SspA), which represented the largest recurrent gene-level overlaps. Each gene was mutated in five independent 4Fi–7Fi lineages, implicating translational tuning of fluorotryptophan incorporation and stress-response/stringent-response rewiring as major adaptive routes under fluoroindole pressure. Given their high parallelism and predicted functional relevance, both genes were subjected to experimental validation in subsequent sections. A secondary convergence tier involved *rpoB*/*rpoC*, encoding core RNA polymerase (RNAP) subunits, and *metK*, encoding *S*-adenosylmethionine (SAM) synthetase, each mutated in four lineages, pointing to reproducible selection on global transcriptional reprogramming^36,37^ and SAM-dependent regulation to mitigate metabolic and proteotoxic stress.

These targets and the remaining recurrent mutated genes are mapped to broad functional modules spanning stress signaling, translation/proteostasis, information processing, envelope/transport functions, central metabolism, and RNA regulation or poorly characterized functions (Fig. 3). Together, these intersections define a limited and reproducible set of genetic targets repeatedly selected across otherwise independent evolutionary trajectories.

### Structural modeling of TrpRS adaptation to fluorinated substrates

Adaptation to fluoroindoles was consistently associated with mutations in the tryptophanyl-tRNA synthetase (*trpS*), a known target in fluorotryptophan evolution experiments.^19^ In the 4Fi lineage, c.45T > G (p.Glu15Asp) was present in all sequenced samples, whereas 5Fi adaptation yielded c.559T > G (p.Met187Leu) at the final time point. All three independent 6Fi lineages carried the c.80A > C (p.Gln27Pro) substitution, and 6TUBX-MB3 lineage transiently accumulated a synonymous c.73C > T (p.Leu25Leu) change. Although synonymous, this mutation shifts leucine codon usage from the common CUG (48%) to the rarer UUG (13%), knowing to slow translation^38^ and potentially contributing to adaptation.

Because direct biochemical characterization of TrpRS-fluorinated ligand interactions was experimentally constrained by ligand solubility and confounding assay-background effects, we used structure-based modeling, combining *in silico* mutagenesis with active-site docking, to compare plausible binding poses and interaction patterns of the fluorinated ligands (Fig. S3, SI method 3). Docking of fluorinated tryptophans and tryptophanyl-5′-AMP intermediates revealed conserved binding poses across all variants. Hydrogen-bonding networks remained unchanged for E15D and M187L, with only subtle redistribution of hydrophobic contacts within the binding pocket; in contrast, Q27P induces local backbone rigidification that measurably reorganizes both hydrophobic and hydrogen-bonding interactions without creating new first-shell contacts. Notably, none of the adaptive substitutions introduced new substrate-contacting residues. Instead, Q27P, E15D, and M187L altered local packing and backbone geometry, consistent with functional tuning through indirect structural effects rather than active-site remodeling. These modeling results therefore provide a structural rationale for the recurrent *trpS* mutations, but should be interpreted as supportive rather than mechanistically conclusive because they do not directly measure amino acid activation, substrate discrimination, or tRNA charging by the mutant enzymes.

### Loss of *sspA* as a dominant early adaptation event

**Fig. 4:**
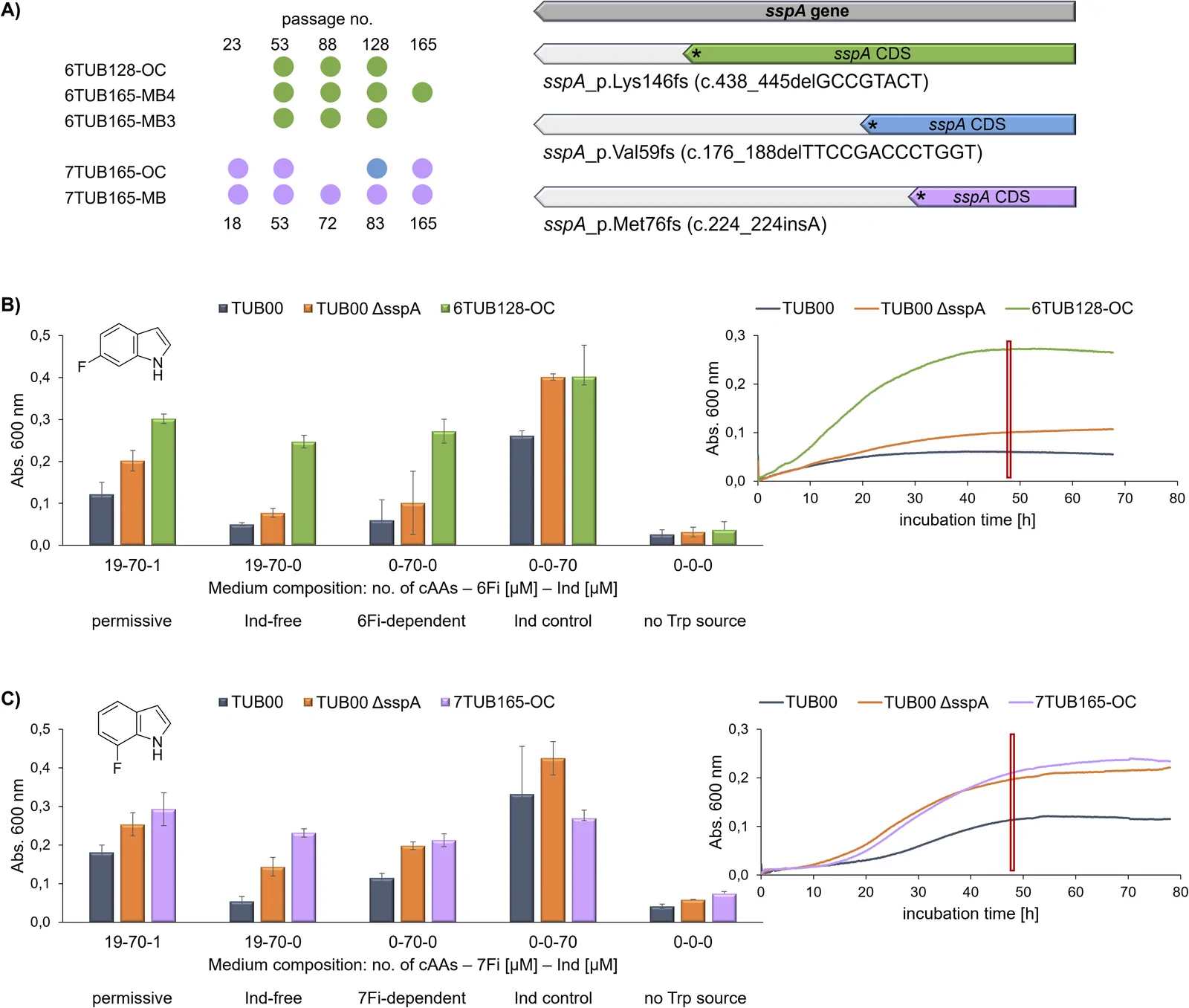
Effects of *sspA* disruption during fluoroindole adaptation. (A) Schematic of the *sspA* locus showing frameshift mutations detected in 6Fi- and 7Fi-adapted lineages. Asterisks denote premature stop codons and shaded regions indicate truncated coding sequences. (B) Growth of ancestral TUB00 (dark blue), Δ*sspA* (orange), and adapted 6TUB128-OC (green) strains in media used during the ALE protocol: NMM19-70-1: ALE starting conditions, NMM19-70-0: end of ALE phase 1, NMM0-70-0: ALE phase 3 (each supplemented with 6Fi), as well as NMM0-0-70 (Ind) as positive control and NMM0-0-0 as negative control. The labels below the *x*-axis indicate the corresponding condition type and stringency. Bar plots show final cell densities after 48 h; corresponding growth curves (NMM0-70-0) are shown at right. (C) Growth of TUB00, Δ*sspA*, and adapted 7TUB165-OC (purple) strains under 7Fi conditions presented as in panel B. Measurements were performed in biological triplicates and repeated three times; the full dataset is provided in Fig. S4A–J.

Genome sequencing identified stringent response regulator *sspA*^39^ as a recurrent and early mutation target in all 6Fi- and 7Fi-adapted lineages. Three independent frameshift mutations were detected: (1) c.176_188delTTCCGACCCTGGT (p.Val59fs), (2) c.224_225insA (p.Met76fs), (3) c.338_445delGCCGTACT (p.Lys146fs), indicating convergent selection at the functional level. All mutations introduced premature stop codons and truncated the 212-aa protein (Fig. 4A). Proteomic analysis (nLC-ESI-MS/MS) confirmed absence of SspA in all adapted strains, while the protein was consistently detected in the ancestral strain and positive controls (ProteomeXchange^40^ PXD048225).^23^

To test functional relevance, *sspA* was deleted in the ancestral strain (TUB00 Δ*sspA*) using CRISPR/Cas9 (SI method 4).^41^ Growth was compared to TUB00 and representative adapted isolates (6TUB128-OC, 7TUB165-OC) across ALE media conditions (Fig. 4B, C and S4A–J, SI method 5). Deletion of *sspA* improved growth in both 6Fi and 7Fi media, particularly in stringent medium (NMM0-70-0). However, the magnitude differed between substrates: (a) 6Fi: Δ*sspA* improved growth but remained below the adapted strain; (b) 7Fi: Δ*sspA* reached growth comparable to the adapted strain.

These results show that loss of *sspA* provides a major fitness benefit during fluoroindole adaptation, but additional mutations with non-additive, background-dependent (epistatic) effects contribute under 6Fi conditions.

## Discussion

Adaptive laboratory evolution (ALE) of *Escherichia coli* on fluorinated indoles reveals an important feature of microbial robustness: cells can survive even substantial perturbation of their core proteome when key regulatory nodes are adjusted. Across four distinct xenobiotic chemistries (4-, 5-, 6- and 7-fluoroindole), our genomic analyses point to a surprisingly coherent evolutionary strategy: (1) attenuation of the stringent stress response *via* disruptive mutations in *sspA* in the 6Fi and 7Fi lineages, and (2) functional refinement of translation through recurrent, structurally meaningful mutations in *trpS*. UpSet analysis highlights convergent adaptation across all four Fi conditions at the level of functional modules, with a small, shared core embedded in a broader, largely lineage-specific mutational background.

### Stress response attenuation: the foundational gateway to a fluorinated proteome

Across all fluoroindole adaptation trajectories, stress-associated regulatory genes emerged as recurrent adaptive targets. Within this broader stress-response axis, disruptive mutation of *sspA* was the earliest and most consistent genetic change in the 6Fi and 7Fi lineages. In all five lineages, independent frameshifts truncate SspA, consistent with a loss-of-function mechanism supported by proteomic data showing that the corresponding protein was no longer detectable. Reconstruction experiments using a *sspA* deletion further demonstrated that loss of SspA alone confers a large fraction of the growth advantage under fluorinated conditions. These findings indicate that the principal barrier to growth therefore lies not simply in the chemistry of fluorotryptophan itself but from the cellular response it provokes.

Fluorotryptophan incorporation likely perturbs proteome stability and triggers a persistent stress signal. Under normal physiology, SspA participates in enforcing the stringent transcriptional program, shifting the cell from growth to survival mode by modulating RNAP activity.^42,43^ Under continuous xenobiotic pressure this response becomes maladaptive: the cell repeatedly activates a survival program in response to a condition that cannot be resolved. Loss of SspA relieves this regulatory checkpoint and allows transcription and translation to proceed despite proteome perturbation.

A similar regulatory streamlining is a common theme in ALE, where attenuating mutations in the global stress regulator *rpoS* (σ^38^)^44^ repeatedly shift investment from stress protection toward growth.^45–48^ Although *sspA* and *rpoS* occupy distinct positions within the regulatory hierarchy, both ultimately reshape global transcriptional responses associated with stringent and general stress programs: *rpoS* mutations blunt σ^38^-dependent stress transcription, whereas *sspA* inactivation removes an upstream brake on σ^70^-driven expression. The recurrent, early loss of *sspA* thus mirrors the classic *rpoS* growth-survival trade-off under persistent proteotoxic pressure.^49^

The adapted state is therefore characterized not by restoration of native proteome quality but by increased tolerance of a perturbed proteome. Rather than repairing the biochemical incompatibility, evolution removes the regulatory feedback that interprets the perturbation as a lethal emergency. The repeated, early fixation of *sspA* mutations therefore identifies stress-response attenuation as key gateway to subsequent adaptation under proteome-wide fluorotryptophan incorporation.

### Tuning the aminoacylation machinery: TrpRS as a convergent adaptive target

Once growth inhibition is relieved, a second constraint emerges: maintaining sufficient translational flux despite incorporation of chemically modified amino acids. This constraint repeatedly targets *trpS*, encoding tryptophanyl-tRNA synthetase. Across independent lineages, the substitutions Q27P, E15D, and M187L recur, yet all map outside the primary substrate-binding shell,^50^ arguing against a classical active-site specificity model and instead suggesting modulation through distal, potentially allosteric effects.^51^ Structural modeling and interaction analysis show preserved ligand orientation and largely intact hydrogen-bonding networks, with exception of Q27P. The mutations therefore do not appear to redesign substrate specificity. Instead, they alter backbone rigidity, packing, and electrostatic coupling, parameters known to influence catalytic cycling and conformational dynamics in aminoacyl-tRNA synthetases. Q27P introduces a helix-breaking proline that constrains the N-terminus and is predicted to bias the conformational ensemble toward catalytically competent states under altered substrate conditions.^52^ E15D subtly modulates electrostatic preorganization, whereas M187L adjusts hydrophobic packing adjacent to the pocket, indirectly reshaping the indole microenvironment without altering binding geometry.^53^

Fluorinated tryptophans are expected to impose a kinetic burden by reducing activation efficiency and increasing misfolding load. Under these conditions, maintaining aminoacylation throughput may become more important than maximizing affinity. Together, the genomic and structural data support a model in which distal mutations bias TrpRS toward productive catalytic states, stabilizing aminoacylation flux under non-native substrates. The enzyme is not made fluorine-specific; it is made operationally robust. This indirect tuning model remains to be tested biochemically, particularly with respect to fluorotryptophan activation, substrate discrimination, and tRNA charging.

Whereas attenuation of the stress response permits continued growth despite proteome perturbation, adaptive tuning of TrpRS supports sustained protein synthesis within the newly established physiological state.

### Lineage-specific remodeling: a hierarchy of adaptive regulators

Beyond *sspA* and *trpS*, additional mutations arose in a lineage-dependent manner, reflecting the different physiological demands imposed by each fluorinated indole (extended results, Tables S6–S11). This produces a layered adaptive architecture. In 4Fi and 5Fi lineages, mutations primarily affected transporters and envelope components, influencing influx, efflux, and membrane robustness. In 6Fi and 7Fi lineages, mutations instead centered on global transcriptional regulators such as RNAP subunits and replication control. These changes appear not to initiate adaptation, but rather to compensate for the physiological imbalance created by proteome-wide substitution.

The adaptation process therefore follows a layered logic (Table 1). Cells first overcome growth limitation, then stabilize translation, while additional mutations subsequently rebalance global physiology in lineage-specific ways. The variation among lineages reflects different compensatory pathways rather than different core solutions.

**Table 1:** Division of labor between the two core adaptive nodes. *sspA* and *trpS* mutations contribute distinct functions to the adaptive phenotype. Additional mutations (RNAP, membrane, transport, metabolism) vary between lineages and likely refine fitness but are not required for the core phenotype

Function	Genetic target	Physiological effect
Remove growth inhibition	*sspA*	Translation continues despite stress
Stabilize translation throughput	*trpS*	Sufficient charging efficiency

### Genetic economy: few mutations, broad physiological effects

Despite the scale of biochemical disturbance, successful adaptation requires remarkably few mutations. Non-hypermutator lineages frequently achieve stable growth with fewer than forty genetic changes, sometimes far fewer. Elevated dN/dS ratios and transversion-biased spectra indicate strong positive selection acting on specific functional targets rather than diffuse genomic drift.

This economy arises from the architecture of cellular control. Mutations in regulatory hubs propagate system-wide effects. Altering a single node such as *sspA* shifts global transcriptional priorities; modifying *trpS* supports translation across the entire proteome. Additional mutations then fine-tune rather than create the adaptive state. Within this narrative of targeted efficiency, hypermutators represent a secondary, though dramatic, mode of exploration: two 6TUBX-MB lineages independently acquired *mutS* defects and accumulated a high mutational burden.^27,28,54^ Hypermutator lineages add complexity but do not alter the central principle: they explore more sequence space^55^ yet converge on the same targets, and we therefore confine their distinct trade-offs to the SI, with a dedicated analysis planned for future work.

## Experimental

A detailed description of all methods is provided in SI methods 1–5.

### Whole-genome sequencing and variant calling

Population samples were recovered from archived glycerol stocks collected at multiple time points along each adaptive lineage (typically 4–8 passages). Strains were cultured in LB, gDNA extracted, quality-checked by NanoDrop, and Illumina paired-end sequenced (2 × 150 bp). Genomes were assembled with SPAdes (“--isolate”).^56^ An ordered, annotated reference genome (TUB00) was generated by mapping to *E. coli* K-12 MG1655 (NC_000913.3)^57^ in MAUVE/Geneious, concatenating ordered contigs (100 Ns), and annotating with Prokka (*E. coli*, “--rfam”).^58^ Assemblies were mapped to this reference with Snippy (v4.6.0) for SNP calling and effect prediction. 4TUBX/5TUBX DNA was sequenced on an Illumina HiSeq 4000 platform, and reads were mapped to the *E. coli* K-12 MG1655 reference genome (NC_000913.3) for variant calling.

### Variant annotation and mutational analyses

Variants were reported using HGVS nomenclature. Mutational load per passage was visualized as the absolute number of detected variants per time point. For spectra/rate analyses, mutations were aggregated across sequenced time points per lineage, counting each unique mutation once. Mutation rate was calculated as unique mutations per bp per generation and expressed as fold-change relative to W-TUBX. dN/dS was computed from CDS SNPs only; Ti/Tv spectra were derived from all SNPs. Convergent target genes were identified by gene-level aggregation of endpoint mutation sets and temporally persistent fixed variants, followed by set-intersection visualization using the browser-based IntersectMe web tool.

### *In silico* mutagenesis and docking (TrpRS)

TrpRS models were based on PDB 8i1w (chain B; ligand removed) and a Swiss-Model (5v0i). Mutations (E15D, Q27P, M187L) were introduced in PyMOL. Ligands were fluorinated (H → F), minimized (Avogadro),^59^ and docked with PyRx/VinaWizard^60^ (rigid receptor/flexible ligand; active-site constrained). Interactions were compared using LigPlot-style fingerprints.^61^

### CRISPR/Cas deletion of *sspA* and growth assay

*sspA* was deleted in TUB00 using CAGO (pCAGO: universal gRNA target, Cas9, *λ*-Red).^41^ A three-fragment editing cassette (homology arms + CmR-N20PAM) was assembled (Golden Gate),^62^ introduced by electroporation, and verified by colony PCR/sequencing; marker excision and plasmid curing were performed as described. Growth was measured in NMM with defined indole/fluoroindole supplements in 96-well plates at 30 °C with continuous shaking (OD_600_ every 10 min), in biological triplicates and repeated three times.

## Conclusions

Adaptive laboratory evolution of *Escherichia coli* on fluorinated indoles reveals a common evolutionary framework rather than a single invariant adaptive trajectory. Across all four fluorinated indoles, successful adaptation consistently involves coordinated adjustment of stress-response regulation and translational control, although the relative contribution and temporal order of these processes depend on the specific fluorinated substrate. In the 6Fi lineages, early inactivation of *sspA* was followed by recurrent *trpS* mutations, whereas 7Fi adaptation was dominated by early loss of *sspA* without detectable *trpS* adaptation, and 4Fi/5Fi adaptation relied primarily on *trpS* mutations in the absence of recurrent *sspA* disruption. Despite these distinct trajectories, all lineages converge on the same functional outcome: sustained growth despite proteome-wide incorporation of chemically modified amino acids.

Our findings demonstrate that fluorinated indoles do not become successful tryptophan surrogates because they acquire chemical equivalence to natural amino acids. Rather, adaptation is achieved by reshaping the cellular systems that interpret and process these altered building blocks. Attenuation of maladaptive stress signaling, together with optimization of translational performance, allows the cell to accommodate fluorinated amino acids without requiring the evolution of a fundamentally new fluorine-specific metabolism. Expansion into this new chemical space is therefore accomplished through coordinated regulatory and translational adaptation rather than metabolic innovation.

More broadly, this work identifies adaptation to proteome-wide chemical perturbation as a hierarchical process governed by a small number of highly connected regulatory and translational control nodes. This framework extends beyond fluorinated amino acids and provides a general conceptual basis for understanding, and ultimately engineering, microbial adaptation to non-natural metabolites and chemically expanded proteomes.

## Author contributions

C. T.-K.: conceptualization, data curation, formal analysis, investigation, methodology, validation, visualization of all experiments, writing – original draft; J. M. G.: data curation, investigation, validation of *sspA* deletion and growth assays; J. L.: writing – review and editing; M. C.-H. W.: data curation, formal analysis, investigation, methodology, validation, visualization of structural modeling of TrpRS and writing – review and editing; S. A. W.: data curation, formal analysis, investigation, methodology, validation of genome sequencing, and writing – review and editing; A. A. B.: writing – review and editing; T. S.: project administration of genomics methodology; N. B.: conceptualization, project administration, writing – original draft; B. K.: supervision, project administration, writing – review and editing.

## Conflicts of interest

There are no conflicts to declare.

## Supplementary Material

SC-017-D6SC05637G-s001

## Data Availability

The data supporting this article are available in the supplementary information (SI) and in the European Nucleotide Archive (ENA). Supplementary information: additional experimental and methodological details, including whole-genome sequencing and mutational analysis workflows, qualitative mechanistic modeling, chromosomal deletion of *sspA*, growth assays, supplementary tables and figures, and the compilation and description of recurrent mutations. See DOI: https://doi.org/10.1039/d6sc05637g. Raw whole-genome sequencing data generated in this study for the W-TUBX, Ind-TUBX, 6TUBX-OC, 6TUBX-MB4, 6TUBX-MB3, 7TUBX-OC, and 7TUBX-MB lineages have been deposited in the European Nucleotide Archive (ENA) at EMBL-EBI under accession number PRJEB122864 (https://www.ebi.ac.uk/ena/browser/view/PRJEB122864). The 4TUBX and 5TUBX mutation datasets were previously published by Agostini *et al.*^22^ and are available as part of the SI accompanying the original publication. These datasets were re-analyzed here within the comparative population-genomic framework described in this study.
