# Supplementary material for: Genomic signatures highlight stress-response evasion and translational tuning as key drivers of Escherichia coli adaptation to fluorinated tryptophans
Source: Chem Sci. 2026 Aug 21;17(36):17250–60. doi: 10.1039/d6sc05637g (PMC13496137; doi:10.1039/d6sc05637g)
Supplement: Supplementary file 1 [file SC-017-D6SC05637G-s001.pdf]

Supplementary Information (SI) for Chemical Science.
This journal is © The Royal Society of Chemistry 2026

Supplementary Information

Genomic signatures highlight stress-response evasion and translational tuning as key drivers of *Escherichia coli* adaptation to fluorinated tryptophans

Christin Treiber-Kleinke[a], Jana Madelaine Göller[a], Justin Liba[b], Michael Chun-Hin Wong[b], Silver Anthony Wolf[c], Allison Ann Berger[a], Torsten Semmler[d], Nediljko Budisa[b,*], Beate Koksch[a,*]

[a] Institute of Chemistry and Biochemistry – Organic Chemistry, Freie Universität Berlin, Berlin, Germany

[b] Chemical Synthetic Biology Group, Department of Chemistry, University of Manitoba, Winnipeg, MB R3T 2N2, Canada

[c] Genome Competence Centre (MF1), Robert Koch Institute, Berlin, Germany

[d] Coordination Integrated Genomic Surveillance (IGS), Robert Koch Institute, Berlin, Germany.

*Correspondence to: beate.koksch@fu-berlin.de or nediljko.budisa@umanitoba.ca

Table of Contents of Supporting Information

Materials and Methods:

1. Whole genome sequencing 3

2. Mutational load, spectra and structural analyses 3

3. Qualitative mechanistic modeling: in silico mutagenesis, ligand preparation and docking 4

4. Chromosomal deletion of *sspA* using CRISPR/Cas genome editing 5

5. Growth assay to determine the impact of *sspA* deletion 6

Supplementary Note / Extended Results: Mutation descriptions 7

Supplementary Tables 9

Supplementary Figures 22

References 32

Supplementary Tables are provided before Supplementary Figures to reflect the experimental and analysis workflow.

Supplementary Tables (S1-S11)

Table S1. Overview of whole-genome sequenced samples along the ALE. 9
Table S2. Variant consequence spectrum across ALE endpoint populations. 10
Table S3. Complete dataset used to calculate lineage-level mutation rates and fold changes for all ALE populations. 11
Table S4. Complete dataset underlying dN/dS and Ti/Tv calculations across ALE lineages. 12
Table S5. Gene sets used for UpSet analysis of convergent mutations across Fi-adapted ALE lineages. 13
Table S6. Fixed/conserved genetic variations of W-TUBX. 15
Table S7. Fixed/conserved genetic variations of Ind-TUBX. 16
Table S8. Fixed/conserved genetic variations of 4TUBX. 17
Table S9. Fixed/conserved genetic variations of 5TUBX. 19
Table S10. Fixed/conserved genetic variants of 6TUBX-OC. 20
Table S11. Fixed/conserved genetic variations of 7TUBX-OC and 7TUBX-MB. 21

Supplementary Figures (S1-S4)

Figure S1. Genealogical relationships of evolved lineages 22
Figure S2. Spatial distribution and types of mutations in ALE lineages. 25
Figure S3. Structure-based modeling of TrpRS mutants and their local interaction networks. 26
Figure S4. Growth curves of the sspA growth assay 31

Materials and Methods

Safety Statement: No unexpected or unusually high safety hazards were encountered.

1. Whole genome sequencing

For W-TUBX, Ind-TUBX, 6TUBX-OC, 6TUBX-MB4, 6TUBX-MB3, 7TUBX-OC and 7TUBX-MB, whole-genome sequencing was performed on population samples recovered from archived glycerol stocks collected at multiple time points along each adaptive lineage, typically covering 4–8 passages per lineage (Table S1). Strains were cultured in LB medium, and gDNA was extracted using Wizard® Genomic DNA Purification Kit and stored at -20 °C, if necessary. DNA concentration and quality was measured using a NanoDrop device.

All samples were Illumina paired-end sequenced (2x 150bp). Genomes were assembled using SPAdes (v3.15.3)[1] with the "--isolate" flag. The resulting contig names were shortened using the rename function of BBMap (v38.92). TUB00 (sample 1) was extracted and mapped to the *E. coli* MG1655 reference genome (NC_000913.3)[2] using the MAUVE plugin (v1.1.1) within Geneious Prime (v2020.2.3). Ordered contigs were merged by concatenating them with 100 Ns using the masking function of Geneious. TUB00 was then annotated using Prokka (v1.14.6)[3] with its database settings specified for *E. coli* and enabling the annotation of ncRNAs ("--rfam"). This resulted in an ordered, annotated reference genome, which was utilized for further SNP calling. All assemblies were mapped by Snippy (v4.6.0) to the annotated reference genome (Seemann T (2015) snippy: fast bacterial variant calling from NGS reads https://github.com/tseemann/snippy). SNPs were then called, and their functional impact assessed by the Snippy pipeline. In addition, Snippy was used to generate a core SNP profile. The resulting SNP profiles were analyzed in R (v4.1.1) and visualized using ggplot2 (v3.3.5). Genomic analysis was performed in collaboration with the Robert Koch Institute (Berlin).

Chromosomal DNA from samples of the 4TUBX and 5TUBX[4] lineages was sequenced on an Illumina HiSeq 4000 platform. Reads were mapped to the *E. coli* K-12 MG1655 reference genome (NC_000913.3) for variant calling. Sequencing and variant calling were performed by BGI Hong Kong.

The lineage relationships are summarized in Figure S1 and the spatial distribution and types of mutations in ALE lineages are shown in Figure S2.

Variant annotation and nomenclature. Genomic variants in the adapted strains were annotated according to the HGVS (Human Genome Variation Society) nomenclature, using *Escherichia coli* str. K-12 substr. MG1655 (NC_000913) as reference. Although recent proposals for standardized genome-assembly naming aim to improve cross-study comparability,[5] the HGVS framework remains the most widely recognized and systematic convention for precise variant reporting and was therefore adopted here. Coding DNA changes were denoted by "c." and protein-level effects by "p.", with base substitutions, insertions, deletions, duplications, and frameshifts annotated using standardized HGVS syntax (>, ins, del, dup, fs). All fixed mutations are summarized in Table S6-S11.

2. Mutational load, spectra and structural analyses

Temporal mutation-count heatmap. For each ALE lineage, the total number of detected mutations was determined independently for each sequenced passage. At each time point, all mutations present in the population were counted, irrespective of whether they had appeared in earlier passages or represented newly arising variants. Thus, the heatmap reflects the absolute mutation load per passage rather than cumulative or de novo mutation counts. Empty cells indicate passages for which no whole-genome sequencing data were available. This representation enables visualization of temporal changes in mutation burden across lineages and highlights gradual versus abrupt shifts in genomic variation during adaptation.

Mutation aggregation and variant filtering. For each ALE lineage, mutations were aggregated across all sequenced time points to increase statistical power, as individual samples often contained too few variants for reliable downstream analyses. This aggregated mutation set was used for the calculation of mutation rates (Table S3), dN/dS ratios, and Ti/Tv spectra (Table S4). Each unique mutation was counted only once per lineage, irrespective of how many passages it appeared in, to avoid inflating recurrent variants during lineage tracking.

Mutation rate estimation and fold-change calculation (Table S3). The mutation rate (μ) was defined as the number of accumulated unique mutations per base pair and per generation. It was calculated according to equation (1), where N_{mut} denotes the total number of unique mutations detected in a given lineage, G the total number of generations elapsed until the final sampling point, and L the length of the analyzed genomic space (*Escherichia coli* str. K-12 substr. MG1655, complete genome accession NC_000913: 4641652bp). Fold-changes (FC) in mutation rate were calculated relative to the corresponding control lineage (W-TUBX) according to equation (2).

$$\mu = \frac{N_{mut}}{G \cdot L} \tag{1}$$

$$FC = \frac{\mu_{lineage}}{\mu_{reference}} \tag{2}$$

dN/dS calculation (Table S4). dN/dS ratios (non-synonymous to synonymous substitution rate) were calculated exclusively from SNPs located within coding sequences (CDS), because synonymous and non-synonymous substitution rates are defined only for codon-encoded positions. Intergenic mutations, non-coding variants, and frameshift-inducing indels were excluded, as these cannot be classified within codon-based substitution models and would distort selection estimates. This CDS-only approach is standard for genome-wide dN/dS estimation.[6]

Transition/transversion (Ti/Tv) spectra (Table S4). Ti/Tv ratios were computed from all SNPs per lineage. Transitions (Ti) were defined as purine ↔ purine (A ↔ G) or pyrimidine ↔ pyrimidine (C ↔ T) substitutions, whereas transversions (Tv) included all purine ↔ pyrimidine exchanges (A ↔ C, A ↔ T, G ↔ C, G ↔ T). Ti/Tv spectra provide a sensitive indicator of underlying mutational mechanisms, including stress-induced mutagenesis, oxidative DNA damage, and mismatch-repair deficiency.

UpSet plot. To identify adaptive target genes and convergent evolutionary signatures across the Fi-adapted lineages, gene-level mutation sets based on whole-genome sequencing data from all final ALE time points were compiled (Table S5). For each lineage, all non-synonymous, frameshift, indel, stop-gained/-lost, and regulatory-region mutations detected in the endpoint population were included. Synonymous mutations were excluded from endpoint datasets unless independently supported by temporal fixation patterns (see extended results, Table S6-S11). To increase robustness and distinguish adaptive drivers from late-arising hitchhikers, particularly in hypermutator backgrounds, additionally mutations that reached fixation (≥ 90% allele frequency or clonal presence) at two or more intermediate time points were incorporated, even if they were not retained in the final population sample. Such temporally persistent mutations were considered high-confidence adaptive candidates and were separately annotated in downstream analyses. All mutation calls were aggregated at the gene level, such that multiple variants within the same gene counted as a single adaptive target.

Intersection analyses across lineages were performed using the UpSet web tool IntersectMe (https://intersectme.leibniz-fli.de/), which visualizes shared and lineage-specific adaptive target genes and enables identification of recurrently mutated loci indicative of convergent evolution.

3. Qualitative mechanistic modeling: in silico mutagenesis, ligand preparation and docking

Structural preparation and mutagenesis (Figure S3): Structural models of *E. coli* tryptophanyl-tRNA synthetase (TrpRS) were based on the crystal structure PDB 8i1w, and a swiss-model using 5v0i as template. For 8i1w, the bound substrate was removed, and only chain B was extracted from the PDB file to generate a docking-ready protein structure. Missense mutations identified in ALE experiments (E15D, Q27P, M187L) were introduced using the Mutagenesis Wizard implemented in PyMOL (Schrödinger, LLC), selecting the lowest-energy rotamer for each substituted residue. The substrate molecule was extracted from the PDB structure (8i1W for tryptophanyl-5′-AMP (TYM), 5v0i for Tryptophan) and chemically modified (H-F mutation) in PyMOL using the built-in editing functions. The modified substrate was subsequently energy-minimized using Avogadro[7] to relieve local geometric strain prior to docking. TYM and Tryptophan variants were docked into the models using Vinawizard in PyRx software.[8] Ligand docking was performed into the TrpRS active site using a rigid-receptor/ flexible-ligand approach. Docking was constrained to the known substrate-binding region to reproduce catalytically relevant poses. Resulting complexes were ranked using the internal scoring function of the docking algorithm. Docking scores were used for relative comparison only and were not interpreted as absolute binding free energy. Protein-ligand interactions were analyzed using LigPlot-style diagrams[9] to identify hydrogen bonds and hydrophobic contacts. These interaction fingerprints were compared across ligands and TrpRS variants to assess conservation or redistribution of contacts upon fluorination or mutation.

4. Chromosomal deletion of *sspA* using CRISPR/Cas genome editing

For chromosomal deletion of *sspA* in TUB00 CRISPR/Cas genome editing, specifically the CAGO technique (CRISPR/Cas9-assisted gRNA-free one-step (CAGO) genome editing) was used.[10] This relies on the use of the pCAGO plasmid, which encodes a universal gRNA, thus no design of specific gRNAs for each target is required. The gRNA targets a universal CRISPR/Cas9 recognition sequence (N20PAM) with minimal sequence homology to the *E. coli* genome to minimize the off-target effects of Cas9. The N20PAM can be incorporated into any editing cassette and is driven by its homologous gRNA provided on the pCAGO plasmid. Also encoded on the pCAGO plasmid is the Cas9 protein for double-strand-break and the λ-Red system (gam, beta, exo) for homologous recombination.

The editing cassette comprised three fragments: 1) L_homo (left homology arm flanking 5’ *sspA*), 2) CmR-N20PAM (chloramphenicol resistance cassette and N20PAM recognition sequence) and 3) Ls_R_homo (right homology arm flanking 3’ *sspA* and a 40-nt sequence of L_homo at 3’ end). The fragments of the editing cassette were amplified using Q5 DNA polymerase in a two-step PCR and assembled in a one-pot Golden Gate Assembly.[11] The following primer sequences were used:

L_homo-fwd: GTCGGTTTCGTGGTGAATATGATTGC,

L_homo-rev (BsaI): GGCTACGGTCTCAGCCATCTGTATGGATTTGTCACAGCTAACACC,

CmRN20PAM-fwd (BsaI): GGCTACGGTCTCATGGCGAAAATGAGACGTTGATCG,

CmRN20PAM-rev (BsaI): GGCTACGGTCTCACTACCTTACTTCGGTTCGATGGACTATTACGCCCC GCCCTG,

Ls_R_homo-fwd (BsaI): GGCTACGGTCTCAGTAGGGACGACGTGGTGTTAGCTGTGACAAATCC ATACAGAGAAAACCTCCAGGTATAG,

L_short - rev CGACATGGTTGAGAAAC.

For the primer design and in silico assembly of the editing cassette, Geneious Prime 2023.2.1 and the NEBridge Golden Gate assembly tool v2.8.3 from New England Biolabs (https://goldengate.neb.com/#!/) were used. Transformation of the editing cassette into recipient strain

TUB00 pCAGO and induction of λ-Red mediated homologous recombination permitted replacement and, thus, deletion of the *sspA* target gene. An overnight culture of TUB00 pCAGO (LB with 100 µg/mL ampicillin) was incubated at 30 °C, 200 rpm until OD_{600} of around 0.2, and then the λ-Red was induced by the addition of 1 mM Isopropyl-β-D-thiogalactopyranosid (IPTG). The induction prior to transformation of the editing cassette ensured sufficient production of λ-Red recombination genes. After further cultivation to OD_{600} of around 0.6, the cells were rendered electrocompetent and resuspended in 100 µL ice-cold 10 % glycerol. Approximately 600 ng of the editing cassette were transformed via electroporation and selected overnight at 30 °C (repA101ts replicon) on an LB-Agar plate containing 100 µg/mL ampicillin (selection marker of the pCAGO plasmid), 37 µg/mL chloramphenicol (selection marker of the editing cassette) and 1 % glucose (to suppress the leaky expression of Cas9 from the arabinose promotor). Homologous recombination of the editing cassette was verified by colony-PCR and sequencing (Microsynth Seqlab GmbH). Genome editing was completed by Cas9-mediated double-strand break by which the chloramphenicol selection marker is excised and homology sequences that previously have flanked the *sspA* gene are recombined by the λ-Red system. Positive clones were incubated for at least 6 h at 30 °C in 2 mL LB with 100 µg/mL ampicillin containing 1 mM IPTG for expression of the λ-Red system and 10 mM arabinose for Cas9 expression, followed by selection on LB agar (100 µg/mL ampicillin) at 30 °C. The removal of chloramphenicol resistance and correct *sspA* deletion was verified by colony-PCR and sequencing. The pCAGO plasmid was cured by incubation at 42 °C (repA101ts replicon).

5. Growth assay to determine the impact of *sspA* deletion

For the growth assay New Minimal Medium (NMM)[12] was used as in the adaptation experiments. NMM compositions is as follows: 7.5 mM $(NH_4)_2SO_4$, 8.5 mM NaCl, 22.5 mM KH_2PO_4, 50 mM K_2HPO_4, 1 mM $MgSO_4$, 20 mM D-Glucose, 1 mg/L Ca^{2+}, 1 mg/mL Fe^{2+}, 10 ng/L trace elements (Cu^{2+}, Zn^{2+}, Mn^{2+}, MoO_4^{2-}), 10 mg/L, 10 mg/L Biotin, 50 mg/L 19 canonical amino acids (except Trp). NMM was differentially supplemented with indole and/or fluorinated indole as follows: NMM(no. of cAAs – conc. of Fi in µM – conc. of indole in µM).

6TUB128-OC and 7TUB165-OC were chose as representatives because a certain genetic volatility due to the *mutS* defect in the hypermutation strains (6TUB165-MB4 and 6TUB165-MB3) was expected, and the 7TUB165-MB lineage illustrated poorer growth behavior.

The ancestral TUB00, the deletion mutant TUB00 Δ*sspA* and 6Fi-adapted 6TUB128-OC were resuscitated in 4 mL NMM19-0-10 (Ind); and 7Fi-adapted 7TUB165-OC must be resuscitated in 4 mL LB at 30 °C, 180 rpm. Cells of 7TUB165-OC were re-grown (1 : 50 inoculation) in NMM0-70-0 (7Fi) twice to remove residual LB and additionally once in NMM19-0-10 (Ind) to set the same starting conditions as with the other samples. Afterward, the cells were washed three times with 1 mL NMM0-0-0 each to remove residual Ind, 6Fi, or 7Fi (2000 g, 3 min, RT), normalized to OD_{600} = 1 (prevention of differences of inoculation volume), and then 198 µL of the respective medium composition were inoculated to OD_{600} = 0.02. The tested media comprised NMM19-70-1 (ALE starting conditions), NMM19-70-0 (end of ALE phase 1), NMM0-70-0 (ALE phase 3), and NMM0-0-70 (Ind) as positive control as well as NMM0-0-0 as negative control. The growth was monitored in 96-well plates (Greiner, flat bottom clear), sealed with the breathable membrane (Breathe-Easy sealing membrane, Sigma Aldrich), at 30 °C with continuous shaking using a microplate reader (Tecan Infinite M200) that measured the absorbance at 600 nm in 10 min intervals. The measurements were performed in biological triplicates and repeated three times (Figure S4).

Supplementary Note / Extended Results: Mutation descriptions

Here we provide a comprehensive overview of genomic variation across all adaptive laboratory evolution lineages to complement the condensed presentation in the main text. This section summarizes lineage-specific and recurring mutations, with curated full variant tables provided for reference, to enable detailed comparison of evolutionary trajectories without interrupting the narrative flow of the manuscript. Raw whole-genome sequencing data generated in this study for the W-TUBX, Ind-TUBX, 6TUBX-OC, 6TUBX-MB4, 6TUBX-MB3, 7TUBX-OC, and 7TUBX-MB lineages have been deposited in the European Nucleotide Archive (ENA) at EMBL-EBI under accession number PRJEB122864 (https://www.ebi.ac.uk/ena/browser/view/PRJEB122864). The 4TUBX and 5TUBX mutation datasets were previously published by Agostini *et al*.[4]

Genomic variations in W-TUBX, Ind-TUBX. The genomic variations in the positive controls (W-TUBX and Ind-TUBX) reflect general consequences of long-term cultivation and, in the case of the indole control, fluorine-induced effects. Both controls accumulated SNPs diffusely across the genome (Table S6 and Table S7), consistent with weak selective pressure and low-level drift during long-term serial cultivation. A *yibJ* missense mutation (c.97C>A, p.Gln33Lys) appeared transiently in W-TUBX and sporadically in Ind-TUBX; given that *yibJ* encodes a poorly characterized RHS-domain protein YibJ[13] and the variant never fixed, it likely reflects neutral drift rather than adaptive change.

More notably, both lineages showed a shared pattern of recurrent mutations in IS-element transposase genes, comprising four variants in *insAB-1* (missense c.69G>T p.Arg23Ser, synonymous variant c.18_21delATACinsCTAT), and *insA-2* (c.243_249delCCGCCATinsACGTCAC p.Phe81Leu and c.207A>C, p.Thr69Thr). Their repeated occurrence across multiple passages indicates episodic IS-element activation, a signature of genomic plasticity under mild or fluctuating stress. Such activity can restructure local genome architecture and generate compensatory or weakly adaptive variation.[14] Overall, the mutation spectra of W-TUBX and Ind-TUBX are dominated by neutral drift and intermittent IS-element mobilization as characteristic background processes during prolonged cultivation.

Genomic variations in the 4Fi and 5Fi ALEs. Adaptation to 4Fi yielded well-growing cells (4TUB093: generation time of 278min ±18 in NMM with 30µM 4Fi, max. OD_{600} ≈ 1.2).[4] Most mutations in this lineage were missense variants and accumulated late, primarily in isolates 4TUB081 and 4TUB093 (Table S8). The affected genes fall into seven COG-like functional clusters: 1) protein synthesis/translation (*trpS*, *glyQ*, *rpsA*, *rpsJ*), 2) transcription and global stress response (*rpoA*, *spoT*, *cpxA*, *evgS*, *uvrY*), 3) nucleotide metabolism (*nrdB*, dcd, *pyrH*), 4) cell envelope, morphology, division (*mrdA*, *ftsA*, *accA*, *acpT*), 5) aromatic amino acid / Trp precursor pathway (*aroH*, *aroK*), 6) transport and efflux (*mdtF*, *yhjX*, *yfeR*, *frvA*), and 7) carbon/energy and cofactor metabolism (*ispB*, *nanK*, *yjhC*).

In contrast, adaptation to 5Fi progressed more slowly. When amino acids were removed from the medium, cultures reached only modest densities (OD_{600} ≈ 0.4). The final isolate, 5TUB083, retained substantially impaired growth (generation time 1080 ± 26 min in NMM with 30 µM 5Fi; max. OD_{600} ≈ 1.0).[4] Most mutations in this lineage appeared only in the final isolate (Table S9). These changes cluster into 1) transport and efflux (*mdtK*, *mdtO*, *mtr*, *ptsG*), 2) proteostasis and envelope/cell shape (*ftsH*, *mrdA*), 3) DNA interaction/restriction (*mrr*), and 4) redox and cofactor metabolism (*fre*).

A recurring theme across both ALEs is alteration of multidrug efflux systems, which normally mediate detoxification of xenobiotics.[15] Mutations in *mdtF* (c.2276A>G, p.Asp759Gly; in 4TUB049/081/093), *mdtK* (stop gained c.913>T, p.Gln305*), and *mdtO* (c.311A>C, p.Ile104Ser; both in 5TUB083) likely reduce efflux and thereby increase intracellular retention of fluoroindoles because accumulation of this FTrp precursors is crucial for protein synthesis. Conversely, modification of the high-affinity Trp transporter Mtr (*mtr* missense c.280A>G, p.Ser94Pro in all 5TUBX isolates) may adjust uptake specificity or regulation of Trp import. Regulation of Trp biosynthesis diverged between the two ALEs. All 5TUBX isolates carried a *trpR* missense mutation (c.250C>T, p.Arg84Cys), predicted to weaken

operator binding and thereby derepress the trp regulon, potentially increasing flux through tryptophan biosynthesis and 5FTrp formation.[16] In contrast, *trpR* remained wild-type in all 4TUBX isolates.

Genomic variations in 6Fi ALEs and emergence of hypermutators. Adaptation to 6Fi yielded highly fit populations across all three lineages (final populations: growth rate μ ≈ 0.004 min^{-1}, max. OD ≈ 1.8 ±0.19, ≈ 6.1 ±0.8 generation per passage),[17] yet their genomic trajectories diverged substantially. The 6TUBX-OC lineage accumulated only 17 genetic variations, many of which remained conserved across successive passages (Table S10). These mutations are clustered into three functional modules: 1) specific adaptation to 6Fi (*trpS*), 2) global gene expression and stress response (*rpoB*, *pcnB*, *sspA*), and 3) cellular economy and membrane integrity (*can*, *yibW*, *fliL*).

In contrast, the lineages 6TUBX-MB4 and 6TUBX-MB3 evolved into hypermutators, accumulating 175 and 280 SNPs, respectively (see Supplementary Data 1-9). Hypermutation emerged independently between passages 88 and 128, well after the split from their common ancestor. Sequencing of intermediate time points identified mutations in *mutS* (DNA mismatch repair protein MutS) as the initiating events,[18] a frameshift variant c.1057delG p.Ala353fs in MB4 and a missense variant c.515G>C p.Arg172Pro in MB3, consistent with loss of methyl-directed mismatch repair activity. Additional mutations in *mutS* and other DNA repair genes (Gene Ontology GO: 0006281) appeared subsequently, including *mutY*, *polB*, *umuC*, *recN*, and *uvrA* in MB4, and *recQ*, *recN*, *uvrB*, *alkB*, *yedK*, *dinG*, *mutY* and *hchA* in MB3, accompanying stepwise increases in mutation load.[19] These secondary alterations indicate progressive diversification within the mutator backgrounds rather than a single catastrophic failure of repair. Despite the dramatic rise in mutation rate, both hypermutator lineages maintained high fitness under 6Fi selection, suggesting that the adaptive benefits outweighed the cost of accumulating deleterious variants. Notably, hypermutation occurred only in MB4 and MB3, but neither in 6TUBX-OC nor in the more stressed 7Fi-adapted populations, indicating that mutator emergence was not simply triggered by stress intensity but may reflect lineage-specific evolutionary routes. A comprehensive dissection of the hypermutator dynamics and their underlying evolutionary logic will be addressed in a forthcoming publication.

Genomic variations in 7Fi ALEs. The 7Fi-adapted lineages 7TUBX-OC and 7TUBX-MB displayed markedly poor performance throughout the ALE: final growth rates of μ ≈ 0.003 min^{-1}, maximal OD_{600} ≈ 0.5 ± 0.15, and only ≈ 4.6 ±1.2 generations per passage.[17] Both lineages experienced abrupt losses in cell density around passages 72 (7TUB072-MB) and 83 (7TUB083-OC), dropping from OD_{600} ~0.9 to ≤0.3, indicative of severe physiological stress. This phenotype was mirrored by a distinct mutational signature summarized in Table S11. Mutations clustered into four major functional groups: 1) translation and tRNA modification (*ileS*, *dusB*), 2) membrane integrity and metabolite handling (*cdsA*, *brnQ*), 3) stress response and growth regulation (*sspA*, *dnaA*, *msrA*), and 4) transcriptional reprogramming (*rpoB*, *rpoC*).

Two additional mutations directly affected tryptophan metabolism. In *trpR*, encoding the DNA-binding transcriptional repressor that modulates the Trp regulon in response to intracellular Trp levels, a frameshift mutation (c.114_123delCGAAGCGTTG, p.Glu39fs) occurred in strain 7TUB083-OC. The resulting loss of functional TrpR likely caused constitutive *trpBA* expression and uncontrolled 7FTrp production from 7Fi. Its disappearance in subsequent passages suggests that deregulated flux through the Trp pathway may have contributed to the observed fitness collapse around passage 83.[17] In *trpB (*TrpS ß-subunit TrpB*)*, the missense variant c.523C>T (p.Arg175Cys) appeared in 7TUB072-MB and in 7TUB165-MB. Prediction tools such as mutfunc[20] and PROVEAN[21] classify this substitution as functionally impactful, although computational annotation alone cannot fully account for the altered biochemical context of growth on fluorinated substrates.

These genetic changes likely reflect coordinated adaptation to manage the dual challenge to cope with the toxicity and steric clash of 7Fi (due to its unnatural structure/chemistry) and the need to utilize 7Fi as a Trp source (metabolically essential).

Supplementary Tables

Table S1. Overview of whole-genome sequenced samples along the ALE.

	passage number												
**lineage**	**18**	**23**	**34**	**48**	**49**	**53**	**72**	**81**	**83**	**88**	**93**	**128**	**165**
**W-TUBX**	●	●				●	●		●	●		●	●
**Ind-TUBX**	●	●				●	●		●	●		●	●
**4TUBX**			●		●			●			●		
**5TUBX**		●		●					●				
**6TUBX-OC**		●				●				●		●	
**6TUBX-MB4**		●				●				●		●	●
**6TUBX-MB3**		●				●				●		●	●
**7TUBX-OC**	●					●			●				●
**7TUBX-MB**	●					●	●		●				●

Table S2. Variant consequence spectrum across ALE endpoint populations. Absolute counts (abs.) and relative fractions (rel. [%]) of detected variants in each final ALE population (columns), grouped by functional classes (rows: non-coding transcript/misc_RNA, stop-gained/-lost indel/frameshift, synonymous, missense and non-coding). These data form the basis for the mutational landscapes shown in Figure 2A-B.

		non-coding transcript/ misc_RNA	stop lost	stop gained	InDel/ frameshift	synonymous	missense	non-coding
**W-TUB165**	abs.	0	1	0	1	1	2	3
	rel. [%]	0,0	12,5	0,0	12,5	12,5	25,0	37,5
**Ind-TUB165**	abs.	0	1	0	2	2	6	5
	rel. [%]	0,0	6,3	0,0	12,5	12,5	37,5	31,3
**4TUB093**	abs.	0	0	1	2	3	25	1
	rel. [%]	0,0	0,0	3,1	6,3	9,4	78,1	3,1
**5TUB083**	abs.	0,0	0,0	2,0	3,0	1,0	25,0	5,0
	rel. [%]	0,0	0,0	5,6	8,3	2,9	69,4	13,9
**6TUB128-OC**	abs.	0	0	1	2	0	11	3
	rel. [%]	0,0	0,0	5,9	11,8	0,0	64,7	17,6
**6TUB165-MB4**	abs.	2	0	3	32	32	83	23
	rel. [%]	1,1	0,0	1,7	18,3	18,3	47,4	13,1
**6TUB165-MB3**	abs.	3	0	3	41	48	139	46
	rel. [%]	1,1	0,0	1,1	14,6	17,1	49,6	16,4
**7TUB165-OC**	abs.	0	0	1	4	2	11	2
	rel. [%]	0,0	0,0	5,0	20,0	10,0	55,0	10,0
**7TUB165-MB**	abs.	0	1	1	3	0	13	3
	rel. [%]	0,0	4,8	4,8	14,3	0,0	61,9	14,3

Table S3. Complete dataset used to calculate lineage-level mutation rates and fold changes for all ALE populations. For each lineage, the table lists the total number of unique mutations aggregated across all sequenced time points, the corresponding number of generations (end-point samples), the inferred mutation rate (mutations per base per generation), and the fold change (FC) relative to the tryptophan control. These data form the basis for the mutation rate analyses shown in Figure 2D.

lineage	no. mutation	Generation (G)	mutation rate (bp/G)	FC
W-TUBX	22	1273	$3{,}72x10^{-09}$	1
Ind-TUBX	57	1270	$9{,}67x10^{-09}$	3
4TUBX	51	825	$1{,}33x10^{-08}$	4
5TUBX	45	678	$1{,}43x10^{-08}$	4
6TUBX-OC	27	780	$7{,}46x10^{-09}$	2
6TUBX-MB4	357	1024	$7{,}51x10^{-08}$	20
6TUBX-MB3	369	1024	$7{,}76x10^{-08}$	21
7TUBX-OC	27	778	$7{,}48x10^{-09}$	2
7TUBX-MB	32	752	$9{,}17x10^{-09}$	2

Table S4. Complete dataset underlying dN/dS and Ti/Tv calculations across ALE lineages. For each lineage, the table reports the numbers of non-synonymous (dN) and synonymous (dS) substitutions, the resulting dN/dS ratio, the numbers of transitions (Ti) and transversions (Tv), the corresponding Ti/Tv ratio, and the respective sample sizes (n), defined as the number of coding sequences (CDS) for dN/dS and single-nucleotide polymorphisms (SNPs) for Ti/Tv. These data form the basis for the mutation rate analyses shown in Figure 2E.

lineage	n (CDS)	dN	dS	dN/dS	n (SNPs)	Ti	Tv	Ti/Tv
W-TUBX	17	12	3	4.00	22	5	11	0.45
Ind-TUBX	32	16	11	1.45	57	14	30	0.47
4TUBX	40	37	3	12.33	51	28	21	1.33
5TUBX	34	32	2	16.00	45	6	36	0.17
6TUBX-OC	19	16	1	16.00	37	9	15	0.60
6TUBX-MB4	306	184	61	3.02	357	219	54	4.06
6TUBX-MB3	309	193	60	3.22	369	216	68	3.18
7TUBX-OC	22	18	0	NA (dS = 0)	27	4	17	0.24
7TUBX-MB	28	20	2	10.00	32	4	18	0.22

Table S5. Gene sets used for UpSet analysis of convergent mutations across Fi-adapted ALE lineages. The table reports the gene-level targets included in the UpSet input, aggregated from whole-genome sequencing data (multiple variants within the same gene counted once). The Endpoint set genes column marks genes mutated in the final population sample of a lineage (including non-synonymous, frameshift, indel, stop-gained/-lost, and regulatory-region mutations; synonymous variants excluded unless supported by temporal fixation). The Persistent set genes column marks genes supported by temporal fixation within a lineage, defined as detected at ≥2 intermediate time points and/or recurring across passages via independent hits, including cases not retained in the endpoint sample. This data form the basis for the UpSet plot shown in Figure 3.

lineage	endpoint set genes	persistent set genes
4TUB093	*accA, acpT, aroH, aroK, cpxA, dcd, evgS, frvA, ftsA, glyQ, ispB, mdtF, metJ, mrdA, nagZ, nrdB, pykF, pyrH, rpoA, rpsA, rpsJ, speB, speC, spoT, trpS, uvrY, yfeR*	*accA, acpT, aroH, aroK, cpxA, dcd, evgS, frvA, ftsA, glyQ, ispB, mdtF, mrdA, nrdB, pyrH, rpoA, rpsA, rpsJ, spoT, trpS, uvrY, yfeR, yhjX, yjhC*
5TUB083	*cpxA, cra, dhaR, era, fre, ftsH, ileS, insN-2, mdtK, mdtO, metJ, mrdA, mrr, mtr, pntA, ptrA, ptsG, rpoC, speA, topA, tpiA, trpR, trpS, ubiA, viaA, ycgH_1, yehQ, yfbV, yggT, yhcN, yibH*	*fre, ftsH, mrdA, mrr, mtr, ptsG, trpR, trpS, ycgh_1, yehQ*
6TUB128-OC	*can, pcnB, leuS, ybiW, proQ, fliL, ygfB, metK, lptC, sspA, trpS, yibJ, rpoB, rpoB*	*can, pcnB, ybiW, fliL, sspA, trpS, rpoB*
6TUB165-MB4	*yaaU, yacH, can, pcnB, glnD, lpxB, yaiP, ybaL, allE, ybcO, nfrA, nfrB, citC, leuS, gltJ, chiP, kdpD, gpmA, ybiW, ybjS, clpA, cydD, ycbB, elfC, ycbF, rlmI, ymdC, holB, trpA, yciW, fear, maeA, ydeK, marA, ydeE, rsxB, rsxD, ydhX, ydhV, pykF, ydiK, aroH, astE, yeaN, yoaE, proQ, yebA, motA, fliL, fruK, yfaL, ais, arnC, yfbK, yfcC, flk, ypdA_2, hemF, eutA, csiD, emrB, pncC, ascF, mutS, cysI, cysJ, ygcU, xdhA, guaD, speA, metK, yqgF, yghE, yghJ, yqiI, yraK, mtr, sspA, bfr, trpS, yhgE, envZ, bcsC, bcsZ, dppC, eptB, rfaS, ilvB_1, ilvA, rho, yihP, fdoH, fdoG_1, rpoB, dnaB, uvrA, prop, lysU, cadC, lptG, yjhR, fimD, hsdR, mrr*	*rpoB, proQ, pcnB, arnC, leuS, metK, can, trpS, ybiW, sspA, fliL, pykF, trpA, envZ, yebA, speA, ypdA_2, ybaL, yoaE, rlmI, ydhX, mutS, yciW*
6TUB165-MB3	*nhaR, ribF, djlA, rluA, ilvH, cra, pdhR, lpd, can, ldcC, frsA, ykfA, cynR, tauD, rdgC, brnQ, panE, ybaO, acrB, hemH, ybcF, sfmH, ybcH, fepA, entC, pagP, leuS, chiP, ybfF, potE, ybfC, ybgD, gltA, dinG, rhtA, artQ, poxB, clpA, lrp, ycbL, elfC, uup, rlmI, gfcD, insA-2, insAB-1, rutF, rutD, putA, csgA, ymdC, thiK, ymgD, umuC, cvrA, adhE, trpA, yciK, puuP, ydbK, ldhA, nary, narZ, ydeK, lsrB, ydgI, fumC, uidA, add, rsxA, lhr, ydhV, ydiI, ydiK, astA, yeaE, proQ, yecM, fliJ, fliK, cobT, plaP, wbbH, yegE, yegV, yehF, dld, yeiG, fruA, alkB, yfaL, glpT_1, evgS, xapB, dapA, hyfG, yfgD, yfhM, tadA, smpB, emrA, mutS, ygbJ, casA, cysI, ygfM, prfB_2, cmtA, speA, metK, trmI, ysgA_1, yedW_8, yqiG, rpoD, fadH, sstT, yhbQ, lptC, gltF, yhdX, trkA, frlR, php, malP, livK, yhjH, bcsA, bcsF, yhjX, glyS, yiaJ, yibJ,*	*leuS, metK, can, sspA, trpS, ybiW, fliL, rpoB, proQ, fepA, ydeK, fadH, rlmI, insA-2, mutS, insAB-1, pcnB*

	yibH, trmL, spoT, fimZ_7, yidE, dgoR, mioC, gpp, rho, recQ, rhtB, pepQ, yihW, frvX, frvA, rhaD, ptsA, rplL, rpoC, amiB, ytfE, ytfL, nanM, deoA, trpR, yaaJ	
7TUB165-OC	*ileS, cdsA, insAB-1, cysB, metG, yqgE, sspA, dusB, dnaA, metE, sthA, rpoC, mscM, msrA*	*ileS, cdsA, insAB-1, sspA, dusB, dnaA, msrA*
7TUB165-MB	*ileS, brnQ, sdhA, insAB-1, trpB, pgsA, ompC_2, metK, yqgE, sspA, yhgF, yibJ, dnaA, rpoB, rpoC*	*ileS, cdsA, sspA, dusB, dnaA, msrA, brnQ, yibJ, rpoB, rpoC*

Table S6. Fixed/conserved genetic variations of W-TUBX. Recurring genetic variations and/or genes targeted multiple times are listed. Genes encode for: *insA-2*: IS1 protein InsA, *insAB-1*: InsAB' transposase, *infB*: protein chain initiation factor IF2 and *yibJ*: putative Rhs-family protein.

		**passages W-TUBX**							
gene	**Genetic variant**	**18**	**23**	**53**	**72**	**83**	**88**	**128**	**165**
insA-2	missense_variant c.243_249delCCGCCATinsACGTCAC p.Phe81Leu		●						●
insAB-1	synonymous_variant c.18_21delATACinsCTAT p.8		●						●
	missense_variant c.69G>T p.Arg23Ser		●						●
infB	missense_variant c.1828C>T p.Arg610Cys				●				
	disruptive_inframe_insertion c.603_623dupCGTTGCTGAAGAAGCACGTCG p.Arg208_Met209insValAlaGluGluAlaArgArg							●	
yibJ	missense_variant c.97C>A p.Gln33Lys	●	●	●	●		●		

Table S7. Fixed/conserved genetic variations of Ind-TUBX. Recurring genetic variations and/or genes targeted multiple times are listed. Genes encode for: *insA-2*: IS1 protein InsA, *insAB-1*: InsAB' transposase, *rpoS*: RNA polymerase, sigma S (sigma 38) factor, *ptsP*: PTS system, enzyme I, transcriptional regulator (with NPR and NTR proteins), *yibJ*: putative Rhs-family protein and *tdh*: threonine dehydrogenase.

		**passages Ind-TUBX**							
gene	**Genetic variant**	**18**	**23**	**53**	**72**	**83**	**88**	**128**	**165**
insA-2	synonymous_variant c.207A>C p.Thr69Thr		●			●		●	
	missense_variant c.243_249delCCGCCATinsACGTCAC p.Phe81Leu						●		●
insAB-1	synonymous_variant c.18_21delATACinsCTAT p.8						●		●
	missense_variant c.69G>T p.Arg23Ser						●		●
rpoS	disruptive_inframe_deletion c.449_454delTTCGCC p.Ile150_Gln152delinsLys				●				
	frameshift_variant c.12_19delTACGCTGA p.Asn4fs			●					
ptsP	frameshift_variant c.1343_1364delAAGAAAAACTGGGCAGCCGTAT p.Glu448fs								●
	frameshift_variant c.48delA p.Arg17fs							●	
yibJ	missense_variant c.97C>A p.Gln33Lys	●		●					
tdh	missense_variant c.355G>T p.Gly119Cys							●	
	missense_variant c.289C>T p.Arg97Cys								●

Table S8. Fixed/conserved genetic variations of 4TUBX. Recurring genetic variations and/or genes targeted multiple times are listed. Genes encode for: *accA*: acetyl-CoA carboxylase carboxytransferase α subunit, *acpT*: 4'-phosphopantetheinyl transferase AcpT, *aroG*: Phe sensitive phospho-2-dehydro-3-deoxyheptonate aldolase, *aroH*: Trp-sensitive phospho-2-dehydro-3-deoxyheptonate aldolase, *aroK*: shikimate kinase 1, *cpxA*: sensor histidine kinase CpxA, *dcd*: dCTP deaminase, *evgS*: hybrid sensory histidine kinase in two-component regulatory system with EvgA, *frvA*: PTS system fructose-like EIIA component, *ftsA*: cell division protein FtsA, *glyQ*: Gly-tRNA ligase α subunit, *ispB*: octaprenyl diphosphate synthase, *mdtF*: multidrug resistance protein MdtF, *mrdA*: penicillin-binding protein 2 transpeptidase, *nanK*: N-acetylmannosamine kinase, *nrdB*: ribonucleoside-diphosphate reductase 1 β subunit, *pyrH*: uridylate kinase, *rpoA*: DNA-directed RNA polymerase α subunit, *rpsA*: 30S ribosomal protein S1, *rpsJ*: 30S ribosomal protein S10, *spoT*: bifunctional (p)ppGpp synthase/hydrolase SpoT, *trpS*: Trp-tRNA ligase, *uvrY*: response regulator UvrY, *yfeR*: transcriptional regulator of yefH, *yhjX*: uncharacterized MFS-type transporter YhjX and *yjhC*: KpLE2 phage-like element; putative oxidoreductase YjhC.

		**passages 4TUBX**			
gene	**genetic variant**	**34**	**49**	**81**	**93**
accA	missense_variant c.760A>C p.Ile254Leu	●	●	●	●
acpT	missense_variant c.107C>G p.Ala36Gly		●	●	●
aroH	missense_variant c.440T>C p.Val147Ala			●	●
aroK	missense_variant c.197A>G p.Val66Ala			●	●
cpxA	missense_variant c.1064T>C p.Glu355Gly		●	●	●
dcd	missense_variant c.457A>G p.Met153Val			●	●
evgS	missense_variant c.989A>G p.Asp330Gly	●	●	●	●
frvA	missense_variant c.437A>G p.Leu146Pro			●	●
ftsA	missense_variant c.17A>G p.Asp6Gly			●	●
glyQ	missense_variant c.143T>G p.Glu48Ala			●	●
ispB	missense_variant c.418A>C p.Asn140His	●	●	●	●
mdtF	missense_variant c.2276A>G p.Asp759Gly		●	●	●
mrdA	missense_variant c.182A>C p.Leu61Arg	●	●	●	●
nanK	synonymous_variant c.228A>G p.Asn76Asn			●	●
nrdB	missense_variant c.461T>C p.Ile154Thr	●	●	●	●
pyrH	missense_variant c.266G>A p.Gly76Ser	●	●	●	●
rpoA	missense_variant c.791T>C p.Val264Ala		●	●	●
rpsA	missense_variant c.472A>C p.Lys158Gln			●	●
rpsJ	missense_variant c.82T>C p.Thr28Ala			●	●
spoT	missense_variant c.1166A>C p.Tyr389Ser			●	●

trpS	missense_variant c.45T>G p.Glu15Asp	●	●	●	●
uvrY	missense_variant c.274A>G p.Lys92Glu		●	●	●
yfeR	missense_variant c.669A>C p.Ile223Met	●	●	●	●
yhjX	synonymous_variant c.261T>C p.Gly87Gly			●	●
yjhC	synonemous_variant c.900A>G p.Gly300Gly		●	●	●

Table S9. Fixed/conserved genetic variations of 5TUBX. Recurring genetic variations and/or genes targeted multiple times are listed. Genes encode for: *fre*: NAD(P)H-flavin reductase, *ftsH*: ATP-dependent zinc metalloprotease FtsH, *mdtK*: multidrug resistance protein MdtK, *mdtO*: multidrug resistance protein MdtO, *mrdA*: penicillin-binding protein 2 transpeptidase, *mrr*: Mrr restriction system protein, *mtr*: Trp-specific transport protein, *ptsG*: PTS system glucose-specific EIICB component, *trpR*: Trp operon repressor, *trpS*: Trp-tRNA ligase, *ycgH_1*: putative transporter component, N-terminal fragment and *yehQ*: protein YehQ.

		passages 5TUBX		
gene	**genetic variant**	**23**	**48**	**83**
fre	frameshift_variant c.107delT p.Leu37fs	●	●	●
ftsH	missense_variant c.1688A>C p.Leu563Arg		●	●
mdtK	stop_gained c.913C>T p.Gln305*			●
mdtO	missense_variant c.311A>C p.Ile104Ser			●
mrdA	missense_variant c.176A>C p.Ile59Ser	●	●	●
mrr	stop_gained c.668G>A p.Trp223*	●	●	●
mtr	missense_variant c.280A>G p.Ser94Pro	●	●	●
ptsG	missense_variant c.749T>G p.Leu250Arg	●		
trpR	missense_variant c.250C>T p.Arg84Cys	●	●	●
trpS	missense_variant c.559T>G p.Met187Leu			●
ycgh_1	missense_variant c.884insT p.Ser298fs	●	●	●
yehQ	missense_variant c.616G>T p.Val206Phe		●	●

Table S10. Fixed/conserved genetic variants of 6TUBX-OC. Recurring genetic variations and/or genes targeted multiple times are listed. Genes encode for: *can*: carbonic anhydrase 2 monomer, *pcnB*: poly(A) polymerase I, *ybiW*: putative pyruvate formate lyase, *filL*: flagellar biosynthesis, *sspA*: stringent starvation protein A, *trpS*: tryptophanyl-tRNA synthetase, and *rpoB*: RNA polymerase (beta subunit).

		**passages 6TUBX-OC**			
gene	**genetic variant**	**23**	**53**	**88**	**128**
can	missense_variant c.167A>G p.Glu56Gly		●	●	●
pcnB	frameshift_variant c.959dupA p.Lys321fs			●	●
ybiW	missense_variant c.829A>T p.Ile277Phe		●	●	●
fliL	missense_variant c.55C>A p.Leu19Met		●	●	●
sspA	frameshift_variant c.438_445delGCCGTACT p.Lys146fs		●	●	●
trpS	missense_variant c.80A>C p.Gln27Pro		●	●	●
rpoB	missense_variant c.443A>T p.Gln148Leu	●	●	●	●
	missense_variant c.3995C>T p.Ser1332Leu		●	●	●

Table S11. Fixed/conserved genetic variations of 7TUBX-OC and 7TUBX-MB. Recurring genetic variations and/or genes targeted multiple times are listed. Genes encode for: *ileS*: isoleucyl-tRNA synthetase, *cdsA*: CDP-diglyceride synthetase, *insAB-1*: InsAB' transposase, *sspA*: stringent starvation protein A, *dusB*: tRNA-dihydrouridine synthase B, *dnaA*: chromosomal replication initiator protein DnaA, *msrA*: methionine sulfoxide reductase A, *brnQ*: branched chain amino acid transporter BrnQ, *yibJ*: putative Rhs-family protein, *rpoB*: RNA polymerase (beta subunit), and *rpoC*: RNA polymerase (beta' subunit).

		**passages 7TUBX-OC**				**passages 7TUBX-MB**				
gene	**genetic variant**	**18**	**53**	**83**	**165**	**18**	**53**	**72**	**83**	**165**
ileS	missense_variant c.1250C>T p.Ala417Val			●						
	missense_variant c.1604T>G p.Phe535Cys		●		●		●	●	●	●
	missense_variant c.196A>C p.Ile66Leu							●		●
cdsA	missense_variant c.209A>C p.Tyr70Ser		●		●		●		●	
insAB-1	synonymous_variant c.18_21delATACinsCTAT p.8			●	●					
	missense_variant c.69G>T p.Arg23Ser			●	●					
sspA	frameshift_variant c.224_225insA p.Met76fs	●	●		●	●	●	●	●	●
	frameshift_variant c.176_188delTTCCGACCCTGGT p.Val59fs			●						
dusB	conservative_inframe_deletion c.928_936delCTGGAGGCG p.Leu310_Ala312del		●		●		●		●	
dnaA	missense_variant c.823A>C p.Ile275Leu			●						
	missense_variant c.773T>G p.Leu258Arg		●		●		●	●	●	●
msrA	missense_variant c.310T>G p.Ser104Ala									
brnQ	missense_variant c.143T>G p.Phe48Cys								●	
	frameshift_variant c.836_855dupGTAGCTT CCTGCTGGCGGCG p.Leu286fs							●		●
yibJ	missense_variant c.97C>A p.Gln33Lys					●			●	●
rpoB	missense_variant c.547T>A p.Trp183Arg									●
	missense_variant c.2041A>C p.Met681Leu							●		●
rpoC	frameshift_variant c.4146_4147dupGC p.Gln1383fs							●		●

Supplementary Figures

Genealogical relationships of evolved lineages

Adaptive laboratory evolution (ALE) experiments were performed with a tryptophan-auxotrophic derivative of *Escherichia coli* K12 MG1655, termed TUB00. Starting from this metabolic prototype (Figure S1), two positive controls were evolved for 165 passages on constant concentrations of either tryptophan (W-TUB165) or indole (Ind-TUB165). Four adaptive laboratory evolution (ALE) experiments were initiated using 4-, 5-, 6-, or 7-fluoroindole (4Fi–7Fi). For the 6Fi- and 7Fi-based ALEs, two different strategies for amino acid removal were applied, termed overall concentration (OC) and metabolic blocks (MB), resulting in distinct descendant lineages. The two different MB lineages of the 6Fi ALE resulted from different routes during the amino acid removal. Passage numbers are indicated in the strain designations.

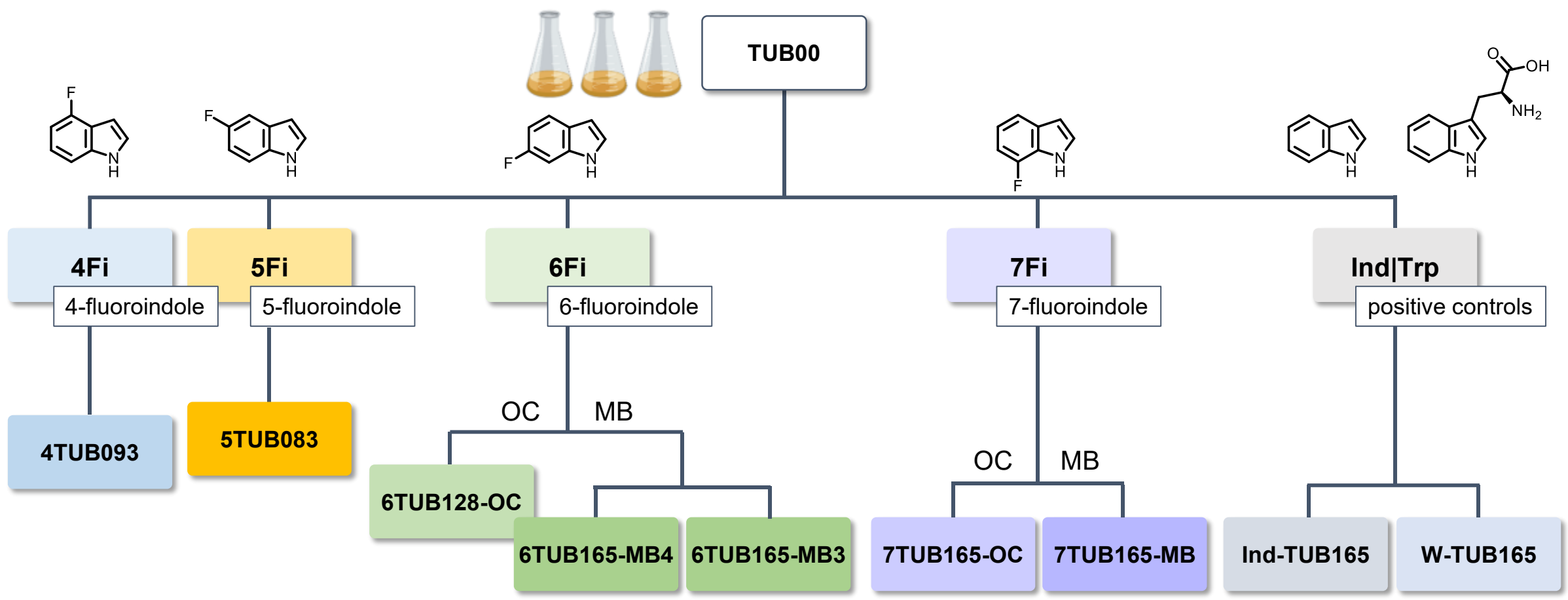

Figure S1. Genealogical relationships of evolved lineages derived from the ancestral strain TUB00 under selection with fluoroindoles (4Fi–7Fi), indole, or tryptophan. OC and MB denote overall concentration and metabolic block amino-acid removal strategies, respectively. Passage numbers are included in strain names.

Genomic mutation landscapes across evolved lineages

To visualize the distribution and nature of mutations acquired during adaptation, we constructed genome maps for each sequenced lineage (Figure S2). Tracks indicate mutation type (missense, synonymous, indel/frameshift, non-coding) and genomic position. Control lineages (W-TUBX, Ind-TUBX) show sparse, randomly distributed variants, consistent with neutral drift during long-term cultivation. Non-hypermutator fluoroindole-adapted lineages (6TUBX-OC, 7TUBX-OC, 7TUBX-MB) display modest mutation loads with clustering in adaptive loci such as *sspA*, *trpS*, and *rpoB*. Hypermutator lineages 6TUBX-MB4 and 6TUBX-MB3 exhibit genome-wide mutation saturation following *mutS* inactivation, though early adaptive targets remain discernible as fixed events preceding the mutator sweep. This spatial overview complements the quantitative mutational spectra presented in Figure 3 of the main text.

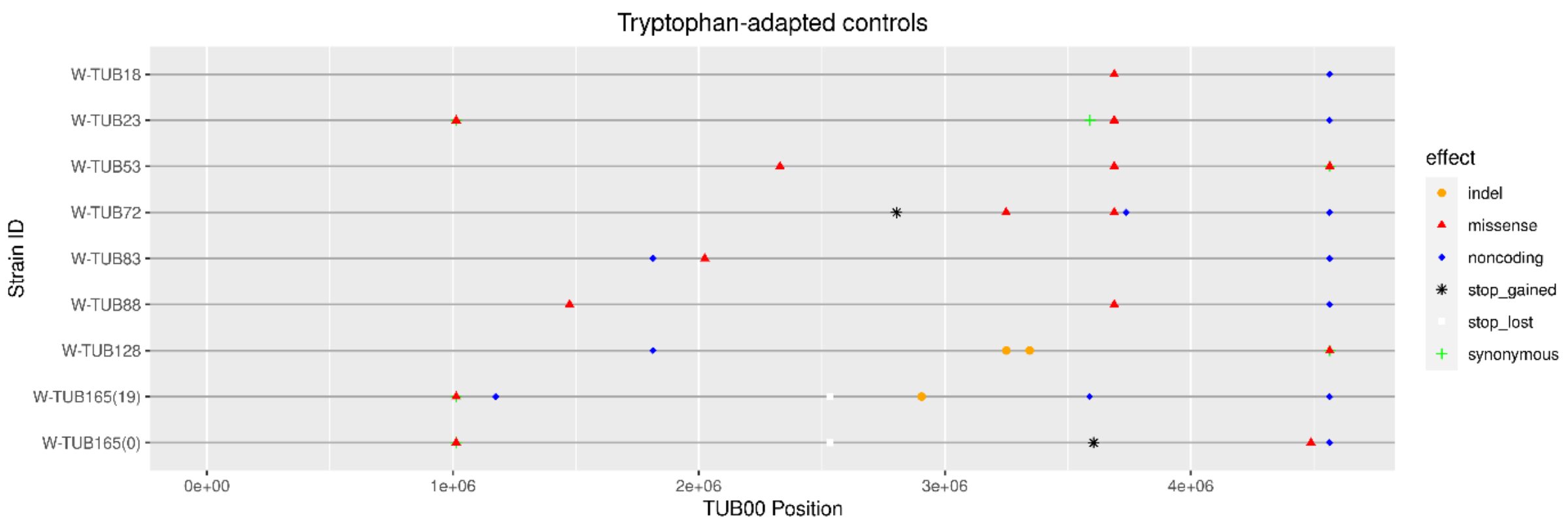

Panel A (W-TUBX): Control lineage evolved on tryptophan. Mutations are sparse and distributed randomly across the genome, consistent with neutral drift during long-term cultivation. No functional clustering is observed.

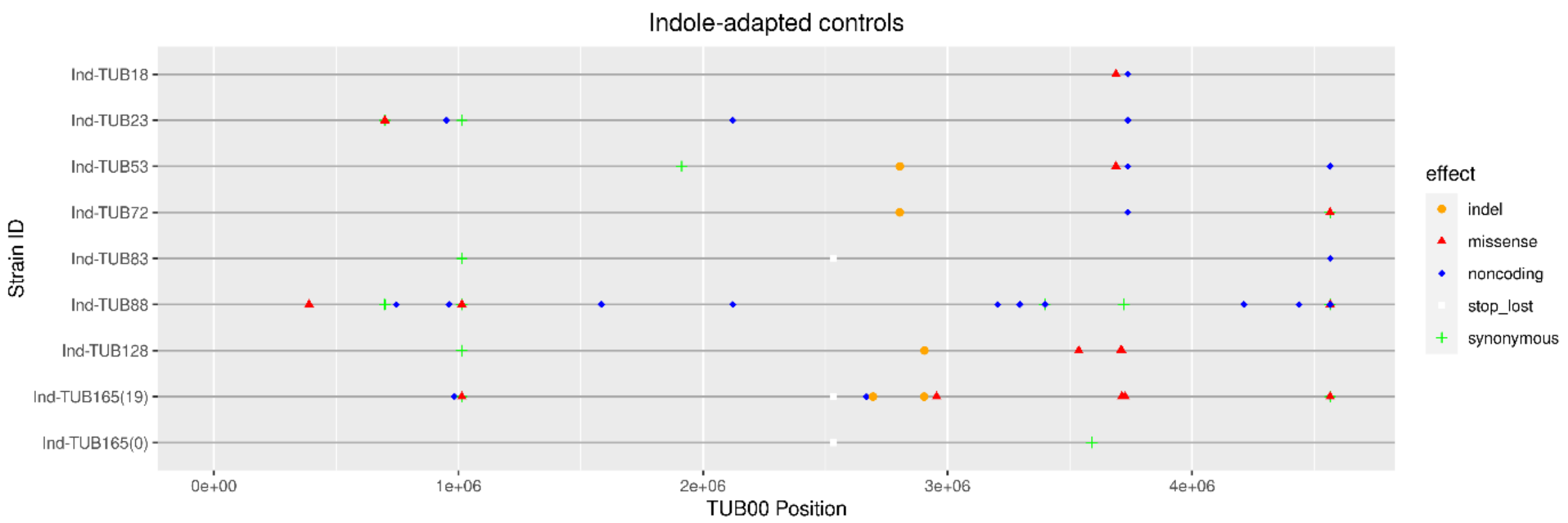

Panel B (Ind-TUBX): Control lineage evolved on indole. Mutation density remains low, though slightly elevated relative to W-TUBX. Mutations appear in isolation without evidence of adaptive sweeps.

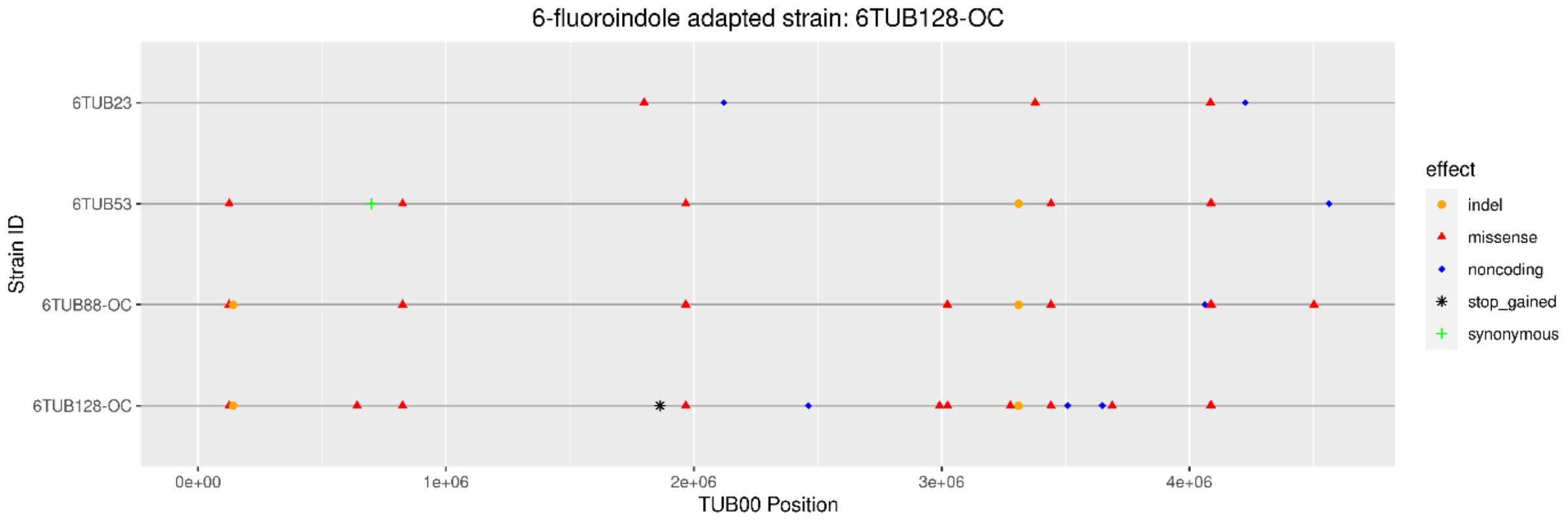

Panel C (6TUBX-OC): 6-fluoroindole-adapted lineage (overall concentration removal strategy). Mutations are modest in number and dispersed, with early fixation of *sspA* and *trpS* variants evident as conserved loci across passages.

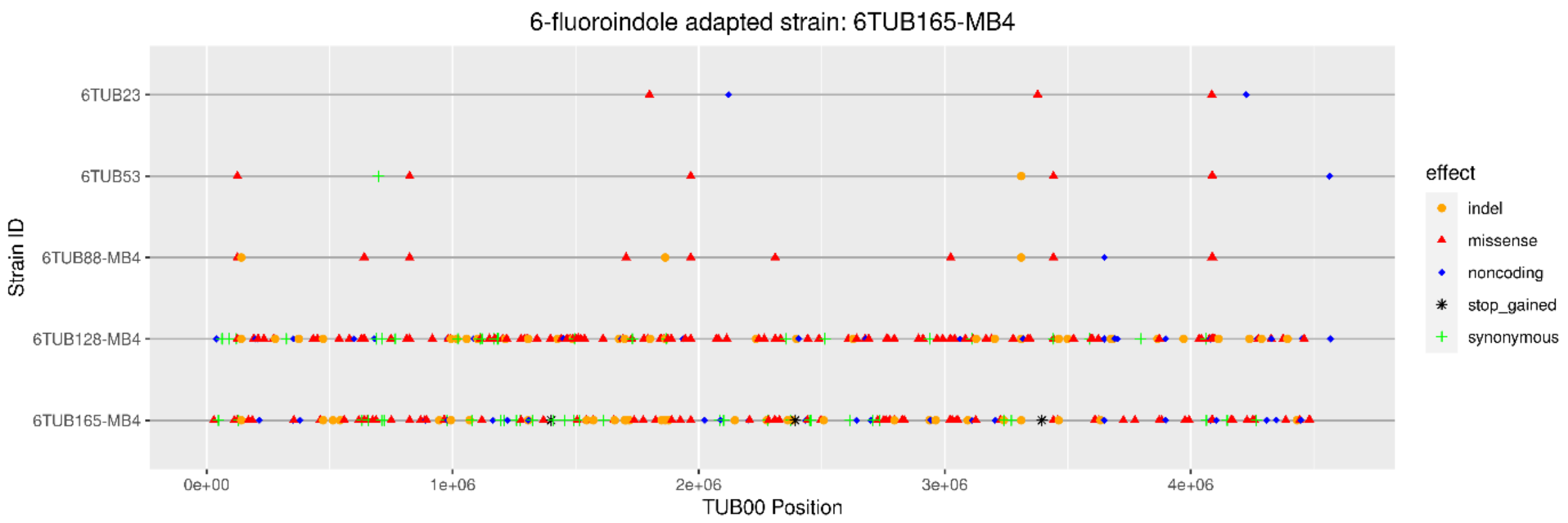

Panel D (6TUBX-MB4): 6-fluoroindole-adapted lineage (metabolic block removal strategy) exhibiting hypermutator phenotype. Mutation density increases dramatically after passage 88, coincident with *mutS* inactivation. Variants spread genome-wide, though adaptive targets (*sspA*, *trpS*, *rpoB*) remain identifiable as early fixed events.

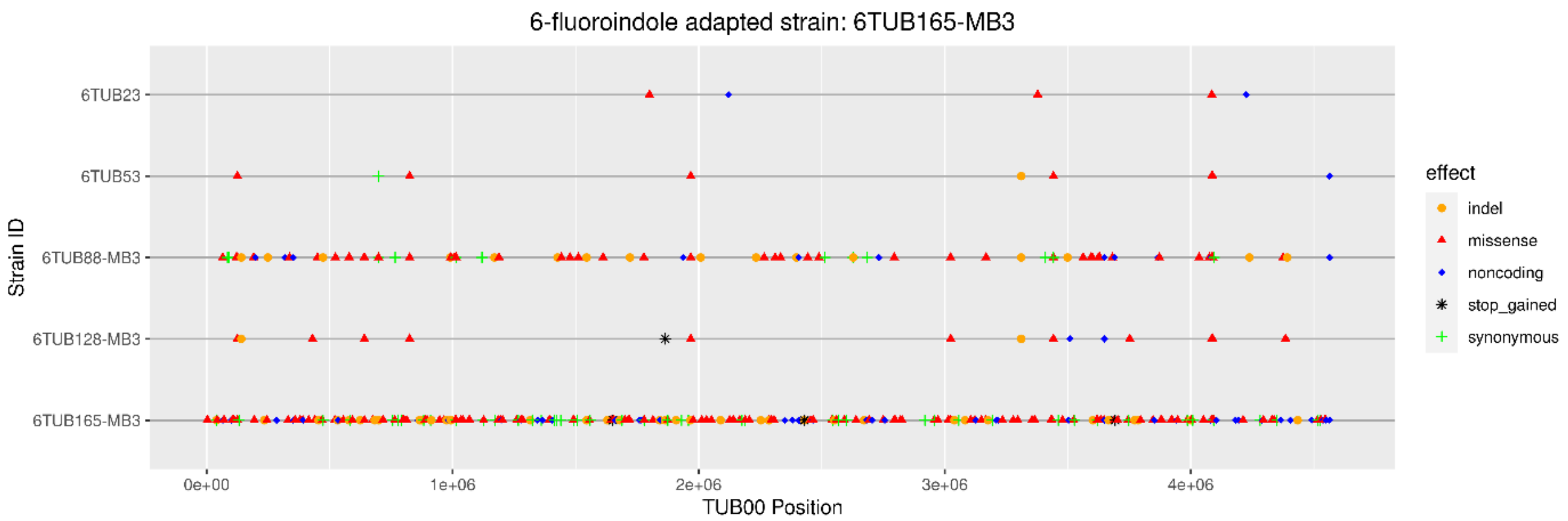

Panel E (6TUBX-MB3): Independent 6-fluoroindole-adapted hypermutator lineage (metabolic block removal strategy). Similar to MB4, abrupt mutation accumulation follows *mutS* disruption, with genome-wide distribution and preserved early adaptive signatures.

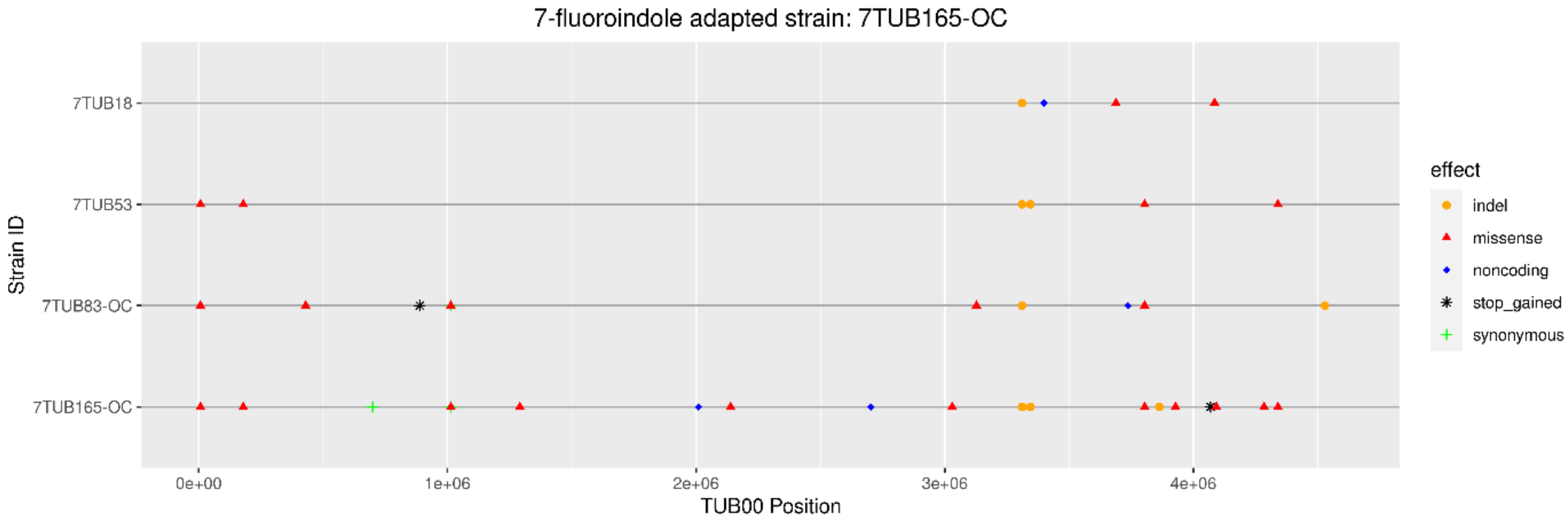

Panel F (7TUBX-OC): 7-fluoroindole-adapted lineage (overall concentration removal strategy). Low mutation burden with clustering in stress-response, translation, and membrane integrity genes. The severe growth phenotype is reflected in the limited adaptive progression.

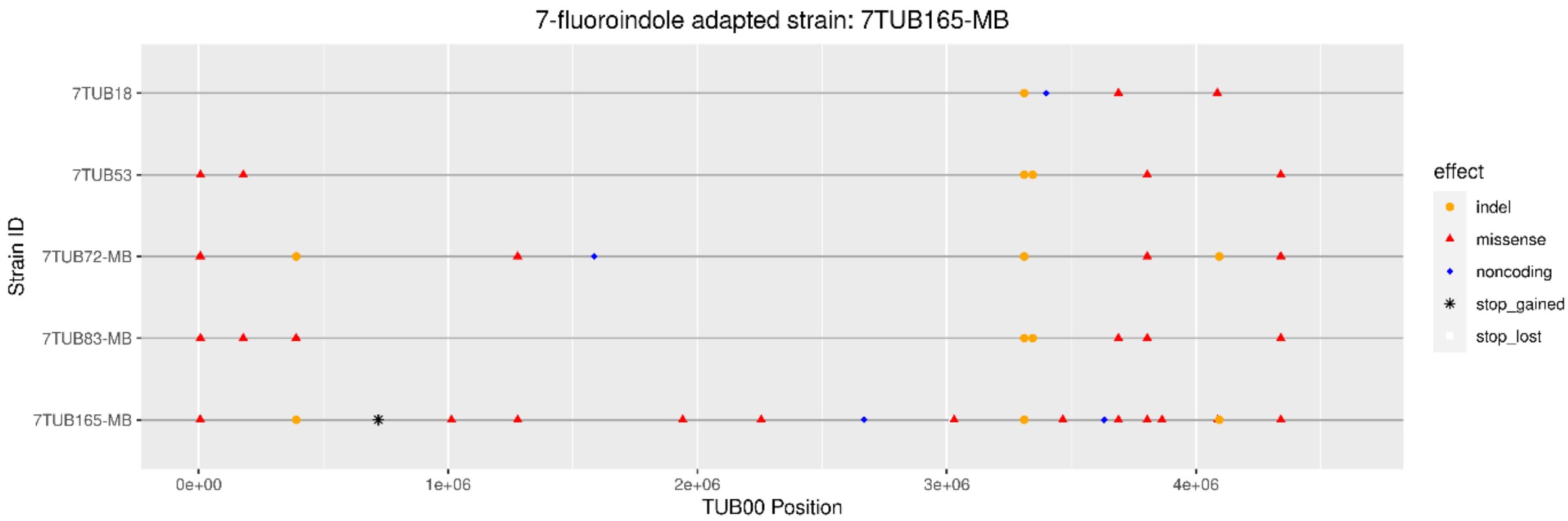

Panel G (7TUBX-MB): 7-fluoroindole-adapted lineage (metabolic block removal strategy). Mutation distribution resembles 7TUBX-OC, with additional variation in transport functions (*brnQ*) and RNA polymerase subunits. The absence of hypermutator emergence despite strong stress highlights lineage-specific evolutionary constraints.

Figure S2. Spatial distribution and types of mutations in ALE lineages. Genome maps (upper to lower: W-TUBX, Ind-TUBX, 6TUBX-OC, 6TUBX-MB4, 6TUBX-MB3, 7TUBX-OC, 7TUBX-MB). Tracks indicate mutation type (missense, synonymous, indel/frameshift, non-coding) and position. Control lineages (W-TUBX, Ind-TUBX) show sparse, randomly distributed variants. Non-hypermutator 6TUBX-OC and 7Fi lineages display modest mutation loads with clustering in adaptive loci (*sspA*, *trpS*, *rpoB*). Hypermutator lineages 6TUBX-MB4 and 6TUBX-MB3 exhibit genome-wide mutation saturation following *mutS* inactivation, though early adaptive targets remain discernible as fixed events preceding the mutator sweep.

Genome maps for the previously reported 4TUBX and 5TUBX lineages can be found in Agostini *et al.* (2021).[4] This spatial overview complements the quantitative mutational spectra presented in Figure 2 of the main text.

Structural Modeling of TrpRS Adaptation to Fluorinated Substrates

Adaptation to fluoroindoles consistently targeted *trpS*. The 4Fi lineage carried p.Glu15Asp, 5Fi acquired p.Met187Leu, and all three 6Fi lineages fixed p.Gln27Pro; one 6Fi lineage also transiently accumulated a synonymous mutation shifting codon usage from common CUG (48%) to rare UUG (13%). Only in the 7Fi lineages *trpS* was not affected by mutation; they exhibit the weakest growth performance among all Fi-adapted strains.

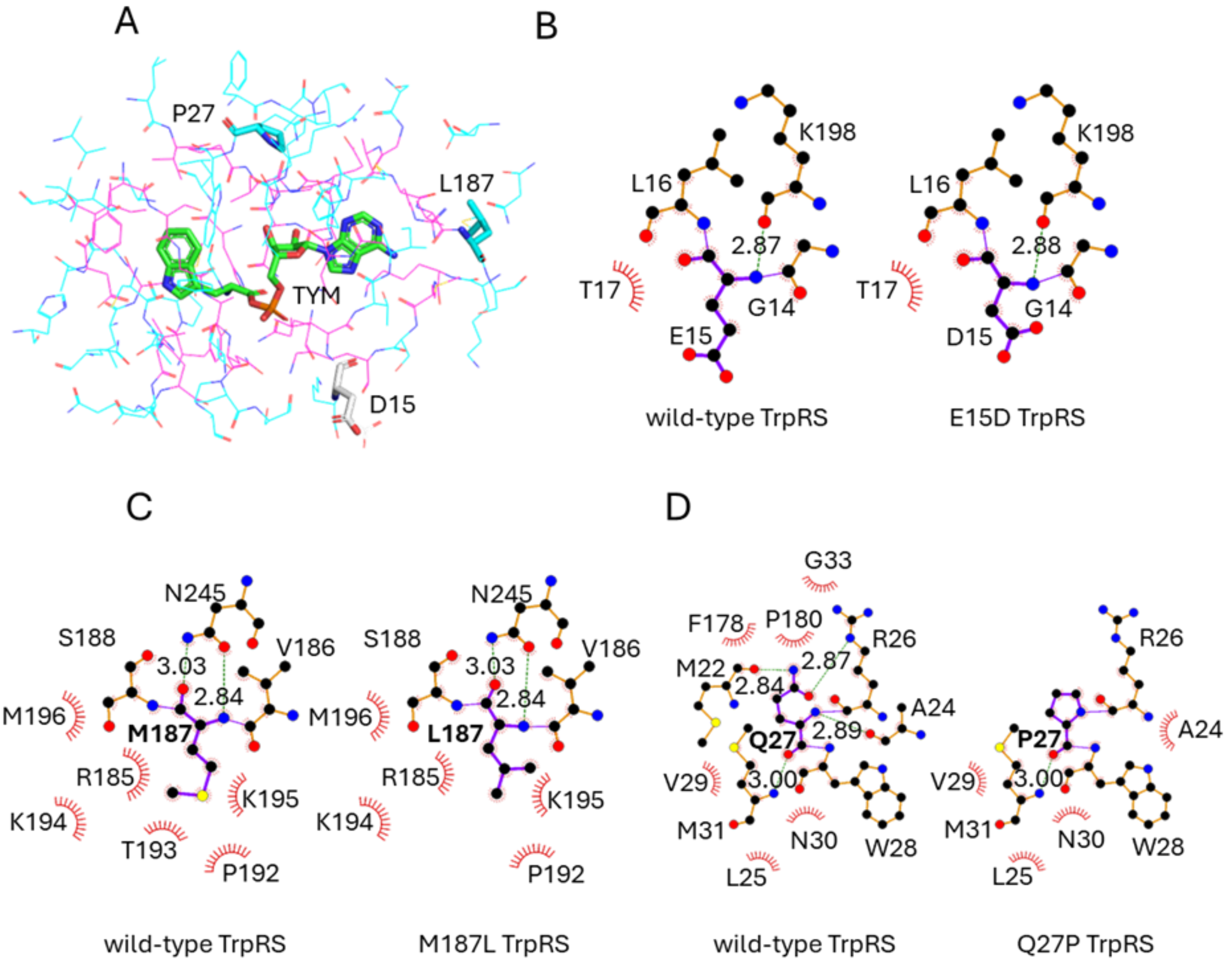

Figure S3. Structure-based modeling of TrpRS mutants and their local interaction networks. LigPlot-style interaction maps illustrating first- and second-shell residues surrounding the substrate-binding region of *Escherichia coli* tryptophanyl-tRNA synthetase (TrpRS) and the positions of adaptive mutations identified by ALE. A) Structural model of wild-type TrpRS (PDB 8i1w) used for docking and interaction analysis, with first- (magenta) and second- (cyan) shell residues are highlighted. B) The E15D substitution represents a conservative, charge-preserving mutation that does not introduce new substrate-contacting interactions and induces only subtle changes in the local electrostatic environment. C) The M187L substitution, located adjacent to but outside the substrate-binding pocket, is accommodated by a modest reduction and redistribution of hydrophobic contacts, resulting in local repacking without perturbation of the overall binding geometry. D) The Q27P substitution, fixed across all 6Fi-adapted lineages, introduces local backbone rigidification that leads to detectable reorganization of hydrophobic and hydrogen-bonding interactions. Importantly, this mutation does not generate new first-shell contacts but instead modulates local packing and interaction topology. Together, these interaction maps demonstrate that adaptive TrpRS mutations predominantly affect distal packing, electrostatic coupling, and backbone geometry rather than directly remodeling substrate contacts, supporting an allosteric tuning model of aminoacylation under fluorotryptophan stress.

Because biochemical characterization was limited by solubility and binding enthalpies, we employed structure-based modeling (Figure S3). Docking revealed conserved binding poses across all variants. Hydrogen-bonding networks remained unchanged for E15D and M187L, with only subtle hydrophobic repacking. Q27P induced local backbone rigidification that reorganized both hydrophobic and hydrogen-bonding interactions without creating new first-shell contacts.

Notably, none of the substitutions introduced new substrate-contacting residues. Instead, all three mutations altered local packing and backbone geometry, indicating functional tuning through indirect structural effects rather than active-site remodeling.

Growth phenotypes upon *sspA* deletion

To assess the functional contribution of *sspA* loss to fluoroindole adaptation, we compared growth of the ancestral strain TUB00, the *sspA* deletion mutant TUB00 *ΔsspA*, and evolved isolates (6TUB128-OC, 7TUB165-OC) across media conditions mirroring the ALE selection regime (Figure S4). All measurements were performed in biological triplicates with three independent repeats; representative curves are shown.

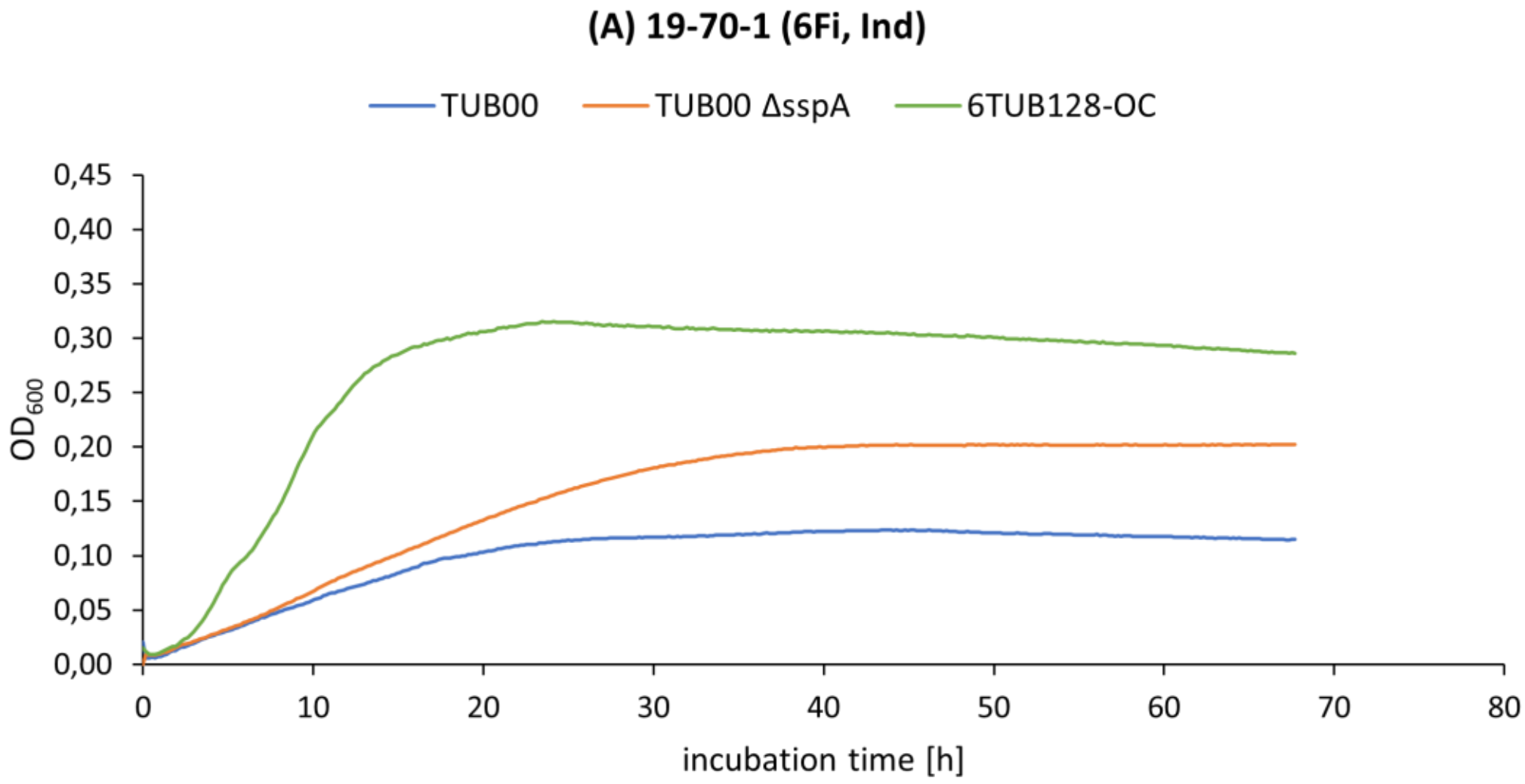

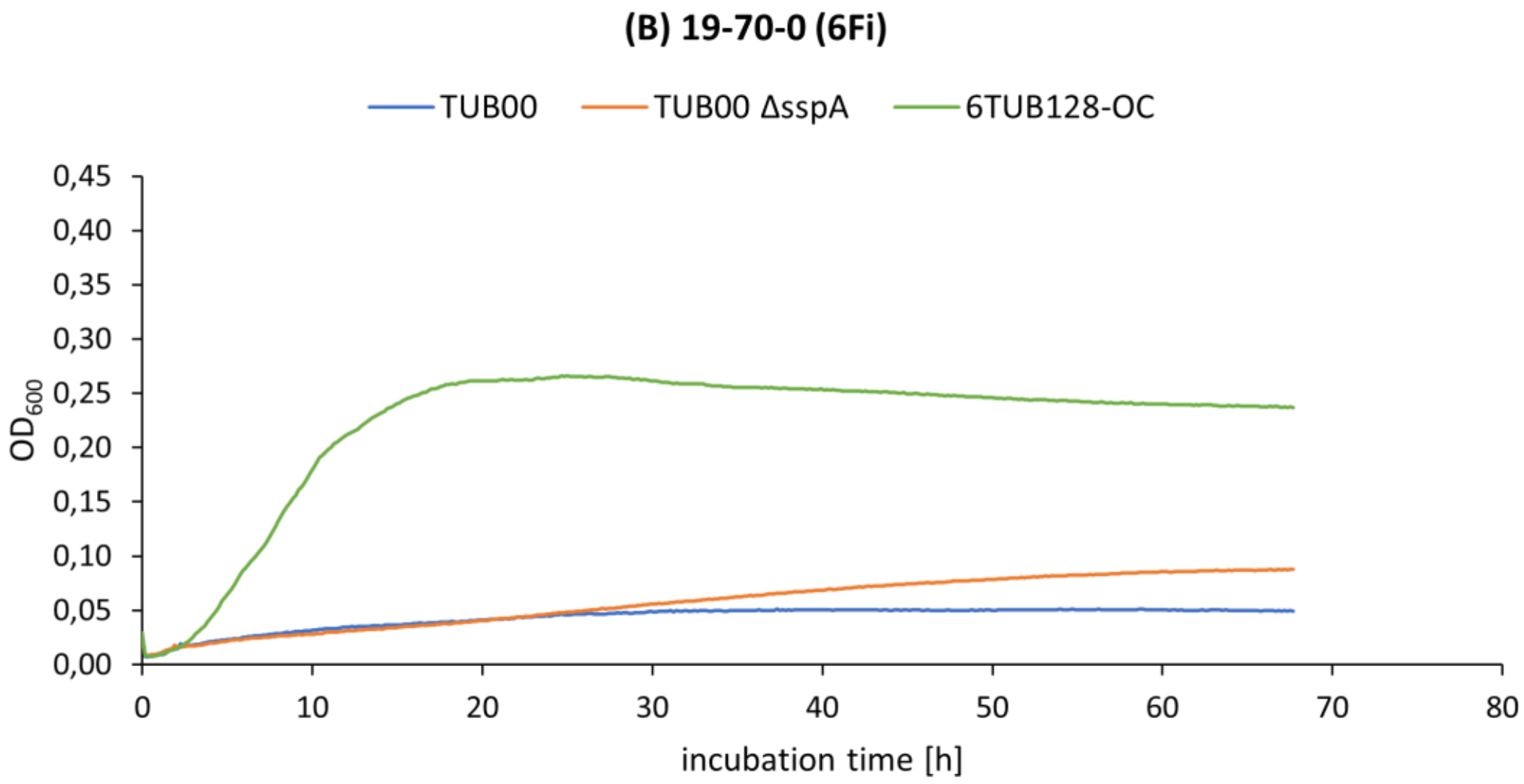

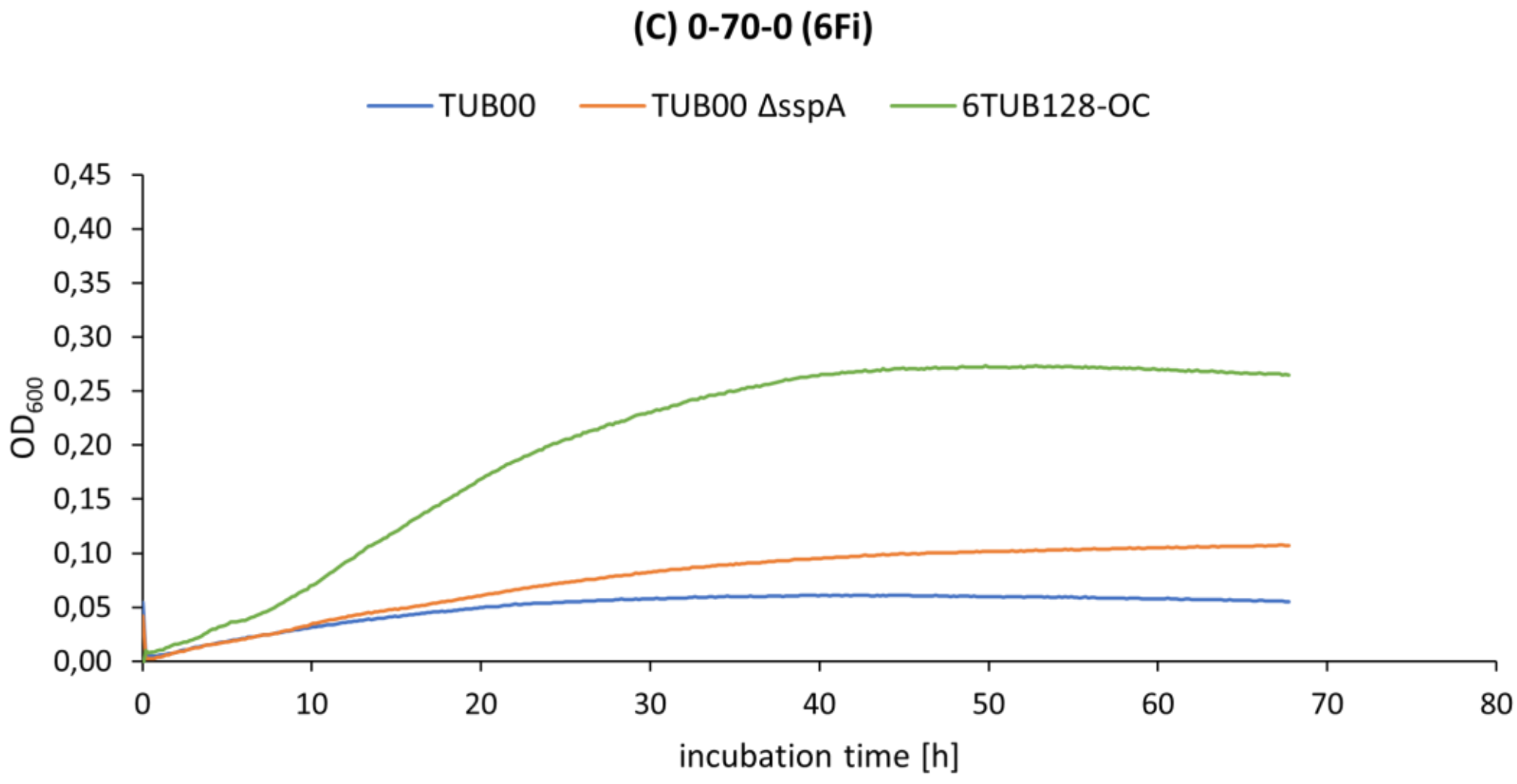

(C) 0-70-0 (6Fi)
TUB00
TUB00 ΔsspA
6TUB128-OC
OD600
0,45
0,40
0,35
0,30
0,25
0,20
0,15
0,10
0,05
0,00
0
10
20
30
40
50
60
70
80
incubation time [h]

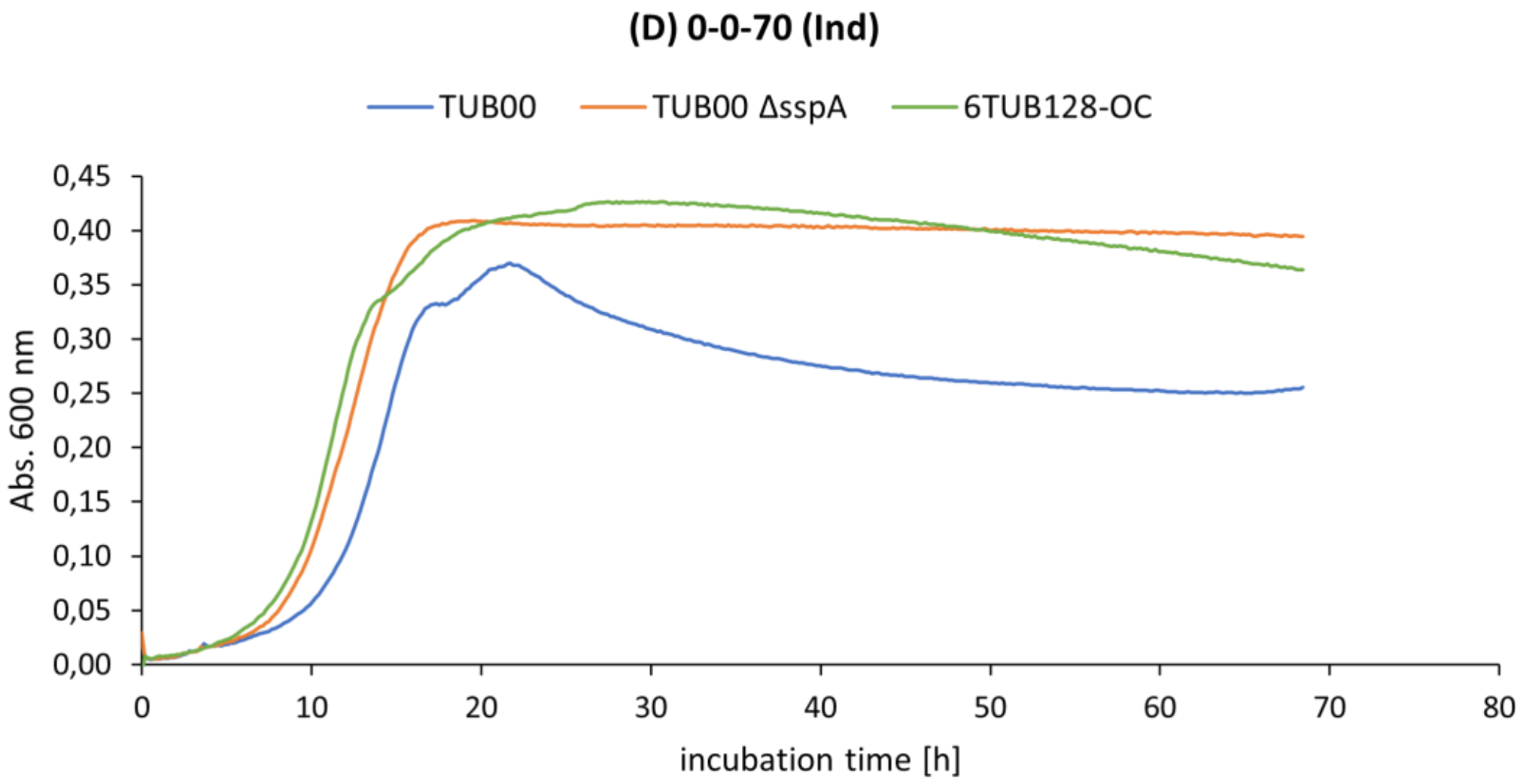

(D) 0-0-70 (Ind)
TUB00
TUB00 ΔsspA
6TUB128-OC
Abs. 600 nm
0,45
0,40
0,35
0,30
0,25
0,20
0,15
0,10
0,05
0,00
0
10
20
30
40
50
60
70
80
incubation time [h]

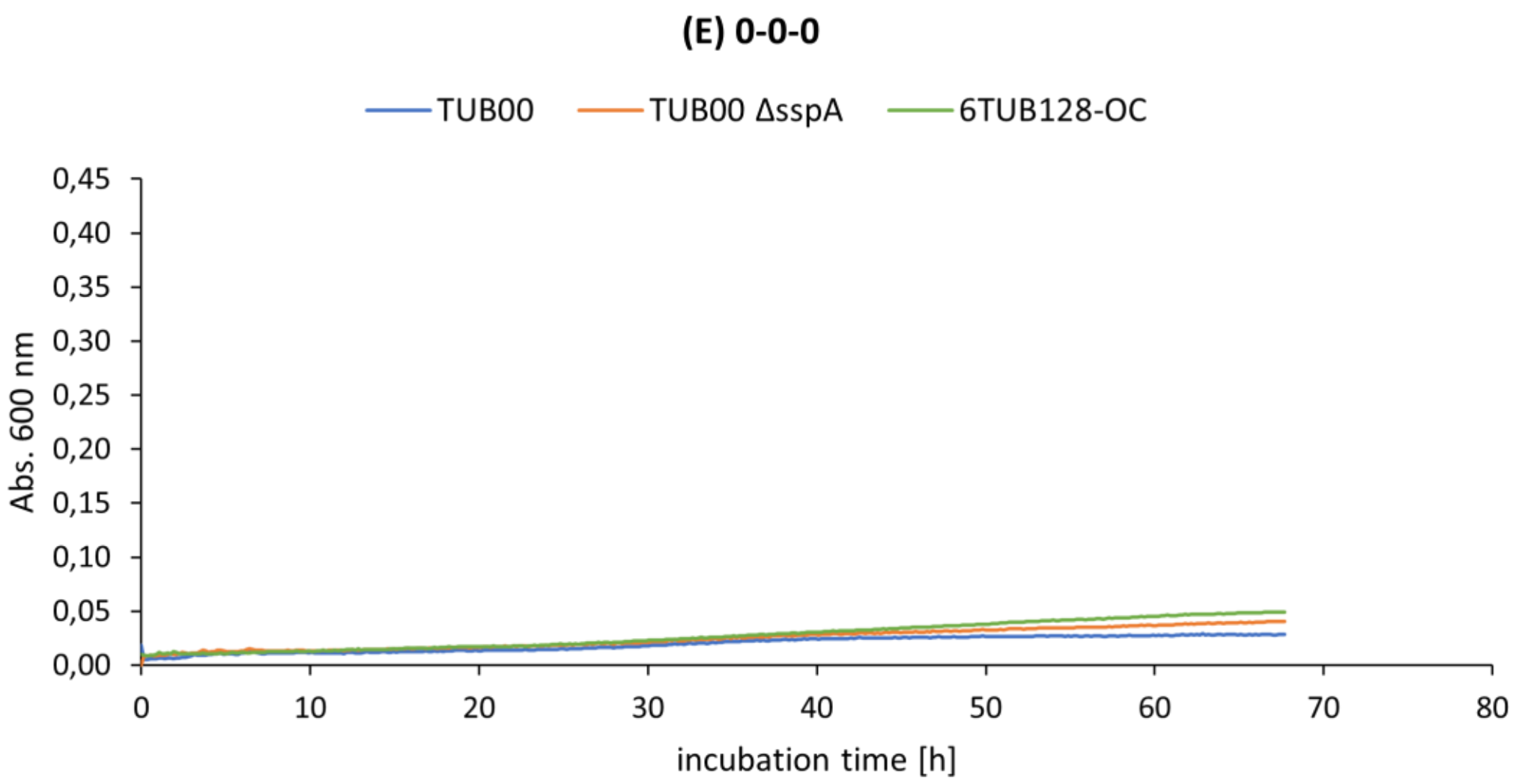

(E) 0-0-0
TUB00
TUB00 ΔsspA
6TUB128-OC
Abs. 600 nm
0,45
0,40
0,35
0,30
0,25
0,20
0,15
0,10
0,05
0,00
0
10
20
30
40
50
60
70
80
incubation time [h]

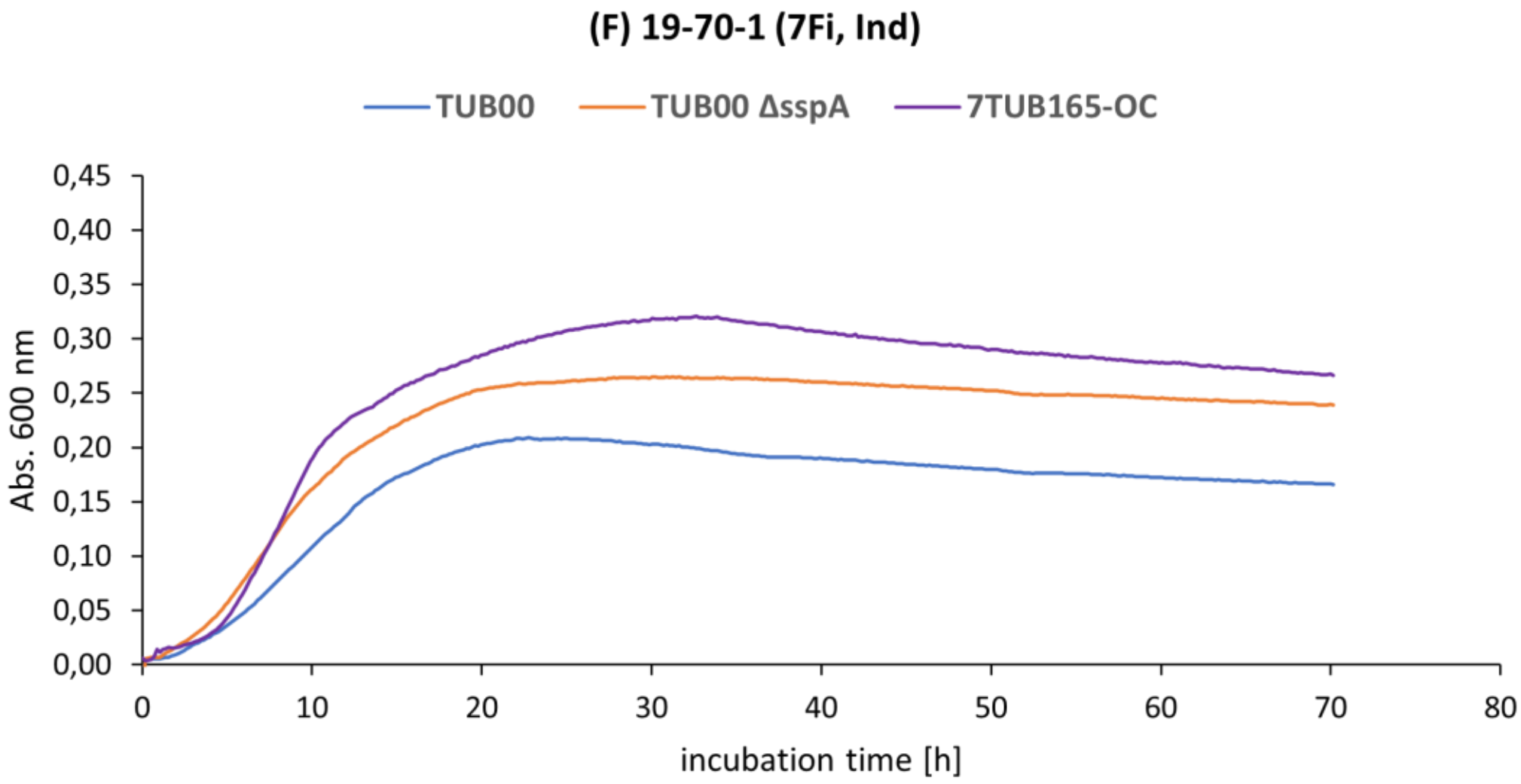
(F) 19-70-1 (7Fi, Ind)
TUB00
TUB00 ΔsspA
7TUB165-OC
Abs. 600 nm
incubation time [h]

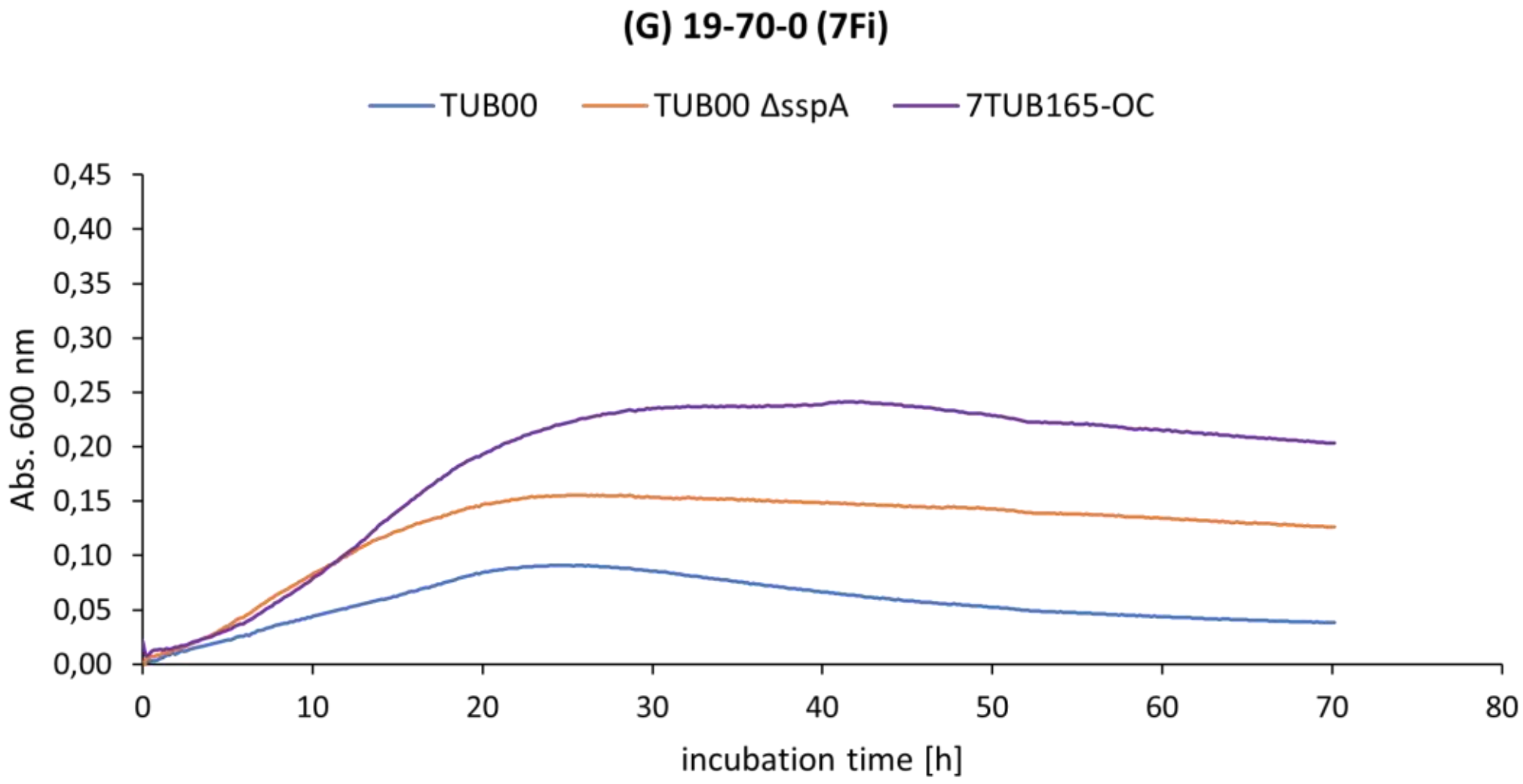
(G) 19-70-0 (7Fi)
TUB00
TUB00 ΔsspA
7TUB165-OC
Abs. 600 nm
incubation time [h]

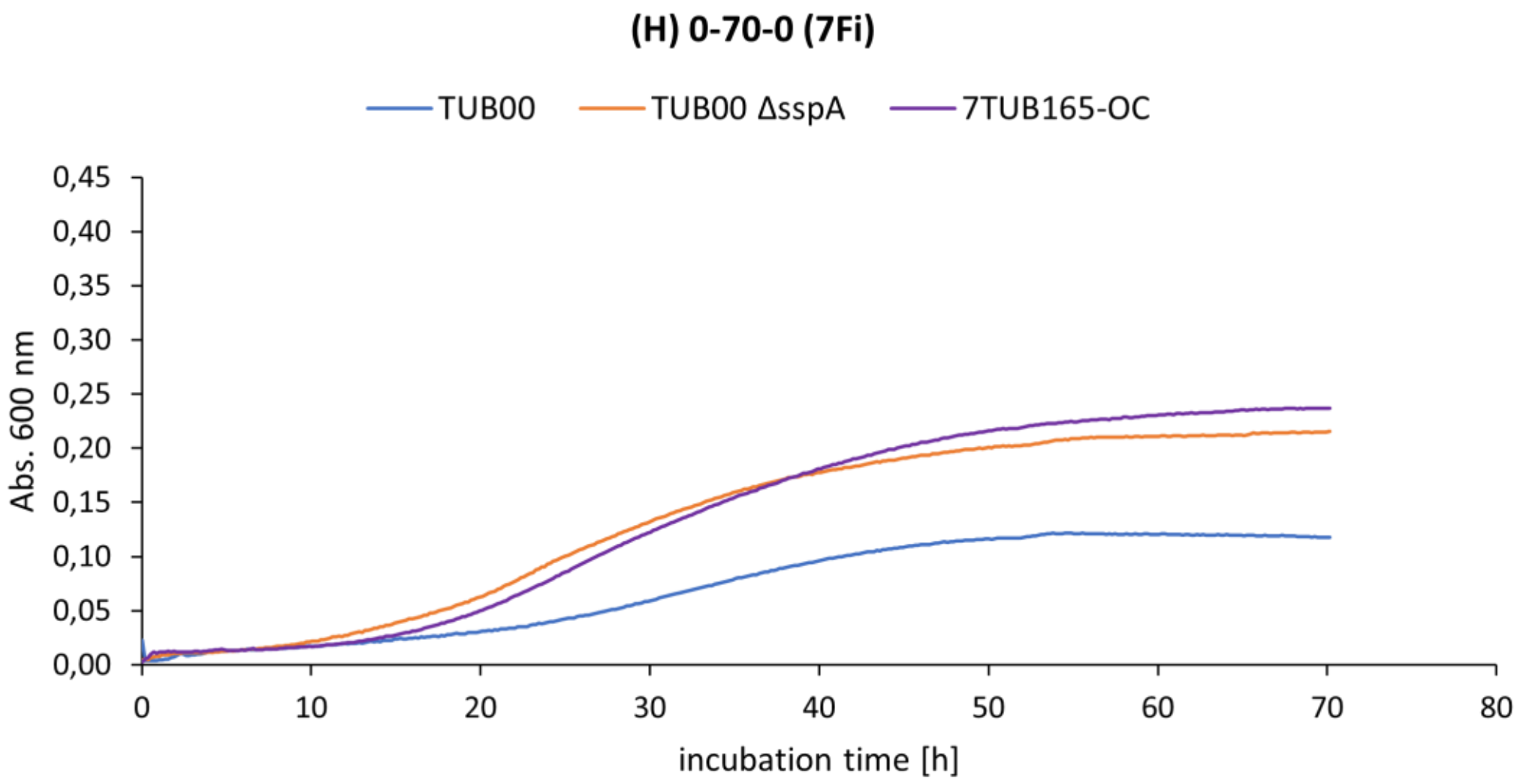
(H) 0-70-0 (7Fi)
TUB00
TUB00 ΔsspA
7TUB165-OC
Abs. 600 nm
incubation time [h]

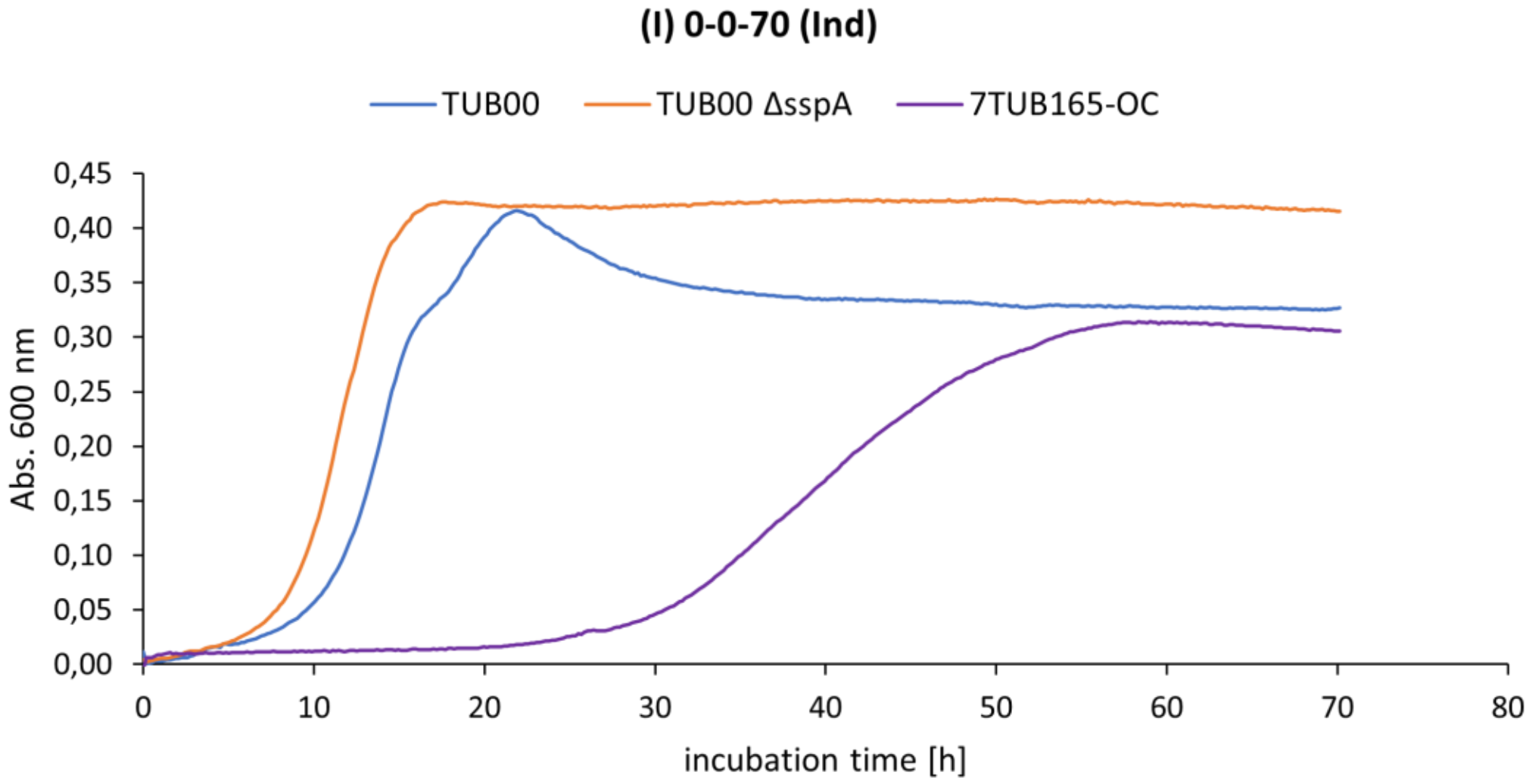

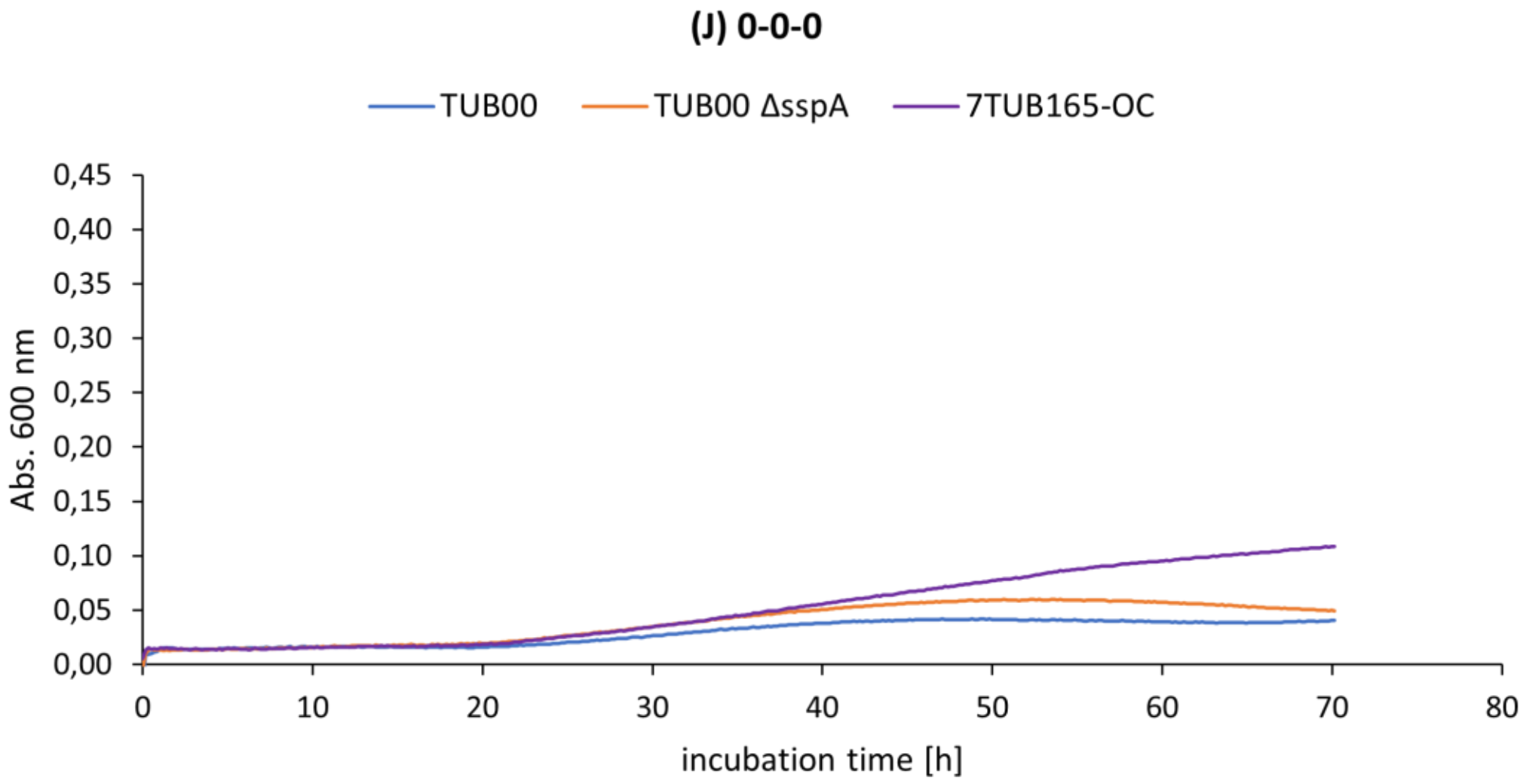

Figure S4. Growth curves of the *sspA* growth assay. In A – E, the curves evaluating the *sspA* impact in 6Fi-supplemented NMM and in F – J those for 7Fi-supplemented medium are shown. The absorption at 600 nm is plotted against the incubation time [h] at 30 °C. Growth curves of TUB00 are colored in blue, curves of the deletion mutant TUB00 Δ*sspA* are orange, curves of 6TUB128-OC in green and curves of 7TUB165-OC in purple. All measurements were performed at microscale level in triplicates using a plate reader. **Panels A–E (6Fi-supplemented media):** Growth in NMM19-70-1 (ALE starting conditions, panel A) shows all strains reach comparable densities, confirming that *sspA* deletion does not impair growth under permissive conditions. Upon indole withdrawal (NMM19-70-0, panel B), *ΔsspA* exhibits improved growth relative to TUB00, though remains below the fully adapted 6TUB128-OC. This advantage becomes more pronounced under exclusive 6Fi reliance (NMM0-70-0, panel C), where Δ*sspA* substantially outperforms the ancestral strain but does not fully recapitulate the evolved phenotype. Positive (NMM0-0-70 with indole, panel D) and negative (NMM0-0-0, panel E) controls confirm strain viability and medium stringency, respectively. **Panels F–J (7Fi-supplemented media):** Under 7Fi, the pattern differs markedly. In NMM19-70-1 (panel F) and NMM19-70-0 (panel G), Δ*sspA* shows growth intermediate between TUB00 and the adapted 7TUB165-OC. However, under exclusive 7Fi reliance (NMM0-70-0, panel H), Δ*sspA* reaches densities comparable to the evolved isolate,

indicating that *sspA* loss alone confers near-complete adaptation to 7Fi. Positive (NMM0-0-70, panel I) and negative (NMM0-0-0, panel J) controls again validate experimental conditions.

Together, these growth profiles demonstrate that *sspA* disruption provides a major fitness benefit under both 6Fi and 7Fi stress, with the effect size being substrate-dependent: partial under 6Fi (requiring additional *trpS* and other mutations for full adaptation), nearly complete under 7Fi.

References

1 A. Prjibelski, D. Antipov, D. Meleshko, A. Lapidus and A. Korobeynikov, *Current Protocols in Bioinformatics*, 2020, **70**, e102.

2 F. Blattner, I. Plunkett G, C. Bloch, N. Perna, V. Burland, M. Riley, J. Collado-Vides, J. Glasner, C. Rode, G. Mayhew, J. Gregor, N. Davis, H. Kirkpatrick, M. Goeden, D. Rose, B. Mau and Y. Shao, *Science*, 1997, **277**, 1453–1462.

3 T. Seemann, *Bioinformatics*, 2014, **30**, 2068–2069.

4 F. Agostini, L. Sinn, D. Petras, C. J. Schipp, V. Kubyshkin, A. A. Berger, P. C. Dorrestein, J. Rappsilber, N. Budisa and B. Koksch, *ACS Cent. Sci.*, 2021, **7**, 81–92.

5 E. K. S. Cannon, D. C. Molk, A. J. Wright, H. Zhang, L. Honaas, K. Chougule and S. Dyer, *GENETICS*, 2025, **229**, iyaf006.

6 Z. Yang and J. P. Bielawski, *Trends in Ecology and Evolution*, 2000, **15**, 496–503.

7 M. D. Hanwell, D. E. Curtis, D. C. Lonie, T. Vandermeersch, E. Zurek and G. R. Hutchison, *Journal of Cheminformatics*, 2012, **4**, 17.

8 S. Dallakyan and A. J. Olson, in *Chemical Biology. Methods in Molecular Biology*, eds. J. E. Hempel, C. H. Williams and C. C. (eds.) Hong, Humana Press, New York, NY, vol 1263., 2015.

9 R. A. Laskowski and M. B. Swindells, *Journal of Chemical Information and Modeling*, 2011, **51**, 2778–2786.

10 D. Zhao, X. Feng, X. Zhu, T. Wu, X. Zhang and C. Bi, *Scientific Reports*, 2017, **7**, 16624.

11 C. Engler, R. Kandzia and S. Marillonnet, *PLoS ONE*, 2008, **3**, e3647.

12 N. Budisa, B. Steipe, P. Demange, C. Eckerskorn, J. Kellermann and R. Huber, *European Journal of Biochemistry*, 1995, **796**, 788–796.

13 P. D. Karp, M. Riley, M. Saier, I. T. Paulsen, J. Collado-Vides, S. M. Paley, A. Pellegrini-Toole, C. Bonavides and S. Gama-Castro, *Nucleic Acids Research*, 2002, **30**, 56–58.

14 J. Vandecraen, M. Chandler, A. Aertsen and R. Van Houdt, *Critical Reviews in Microbiology*, 2017, **43**, 709–730.

15 V. Lázár, I. Nagy, R. Spohn, B. Csörgö, Á. Györkei, Á. Nyerges, B. Horváth, A. Vörös, R. Busa-Fekete, M. Hrtyan, B. Bogos, O. Méhi, G. Fekete, B. Szappanos, B. Kégl, B. Papp and C. Pál, *Nature Communications*, 2014, **5**, 4352.

16 R. Q. Marmorsteins and P. B. Sigler, *The Journal of Biological Chemistry*, 1989, **264**, 9149–9154.

17 C. Treiber-Kleinke, A. A. Berger, L. Adrian, N. Budisa and B. Koksch, *Frontiers in Synthetic Biology*, 2024, **1**, 1345634.

18 A. Jolivet-Gougeon, B. Kovacs, S. L. Le Gall-David, H. Le Bars, L. Bousarghin, M. Bonnaure-Mallet, B. Lobe, F. Guillé, C. J. Soussy and P. Tenke, *Journal of Medical Microbiology*, 2011, **60**, 563–573.

19 B. A. Bridges, *Phil. Trans. R. Soc. Lond.*, 2001, **356**, 29–39.

20 O. Wagih, M. Galardini, B. P. Busby, D. Memon, A. Typas and P. Beltrao, *Molecular Systems Biology*, 2018, **14**, e8430.

21 Y. Choi and A. P. Chan, *Bioinformatics*, 2015, **31**, 2745–2747.
